# A Phase 1 Randomized Placebo-Controlled Study to Assess the Safety, Immunogenicity and Genetic Stability of a New Potential Pandemic H7N9 Live Attenuated Influenza Vaccine in Healthy Adults

**DOI:** 10.3390/vaccines8020296

**Published:** 2020-06-10

**Authors:** Irina Kiseleva, Irina Isakova-Sivak, Marina Stukova, Marianna Erofeeva, Svetlana Donina, Natalie Larionova, Elena Krutikova, Ekaterina Bazhenova, Ekaterina Stepanova, Kirill Vasilyev, Victoria Matyushenko, Marina Krylova, Julia Galatonova, Aleksey Ershov, Dmitry Lioznov, Erin Grace Sparrow, Guido Torelli, Larisa Rudenko

**Affiliations:** 1 Federal State Budgetary Scientific Institution “Institute of Experimental Medicine”, 197376 St Petersburg, Russia; isakova.sivak@iemspb.ru (I.I.-S.); sveta.donina@gmail.com (S.D.); nvlarionova@mail.ru (N.L.); krutikova.iem@mail.ru (E.K.); sonya.01.08@mail.ru (E.B.); fedorova.iem@gmail.com (E.S.); matyshenko@iemspb.ru (V.M.); vaccine@mail.ru (L.R.); 2 Smorodintsev Research Institute of Influenza, Ministry of Health of the Russian Federation, 197376 St Petersburg, Russia; marina.stukova@influenza.spb.ru (M.S.); mariana.erofeeva@influenza.spb.ru (M.E.); kirillv5@yandex.ru (K.V.); office@influenza.spb.ru (D.L.); 3 The Federal State Unitary Enterprise “Scientific and Production Association for Immunological Preparations “Microgen”, Ministry of Health of Russian Federation, 127473 Moscow, Russia; marina.krylova0791@gmail.ru.com (M.K.); yulia.galatonova@gmail.com (J.G.); gcpgmp@mail.ru (A.E.); 4 World Health Organization, 1211 Geneva, Switzerland; sparrowe@who.int (E.G.S.); gtorelli@icloud.com (G.T.)

**Keywords:** H7N9 influenza, potential pandemic vaccine, LAIV, clinical trial, safety, stability, immune response, humoral immunity, cellular immunity

## Abstract

This study describes a double-blind randomized placebo-controlled phase I clinical trial in healthy adults of a new potential pandemic H7N9 live attenuated influenza vaccine (LAIV) based on the human influenza virus of Yangtze River Delta hemagglutinin lineage (ClinicalTrials.gov Identifier: NCT03739229). Two doses of H7N9 LAIV or placebo were administered intranasally to 30 and 10 subjects, respectively. The vaccine was well-tolerated and not associated with increased rates of adverse events or with any serious adverse events. Vaccine virus was detected in nasal swabs during the 6 days after vaccination or revaccination. A lower frequency of shedding was observed after the second vaccination. Twenty-five clinical viral isolates obtained after the first and second doses of vaccine retained the temperature-sensitive and cold-adapted phenotypic characteristics of LAIV. There was no confirmed transmission of the vaccine strain from vaccinees to placebo recipients. After the two H7N9 LAIV doses, an immune response was observed in 96.6% of subjects in at least one of the assays conducted.

## 1. Introduction

The 21st century has seen the emergence and spread of a number of potentially pandemic infectious diseases, such as novel coronaviruses (SARS-CoV, MERS-CoV and SARS-CoV-2) [1] and influenza viruses of swine [2] or avian origin [3].

In March 2013, the first laboratory-confirmed cases of human infection with an Asian lineage of avian influenza H7N9 in two provinces of China, Shanghai and Anhui, were detected and reported to the World Health Organization (WHO) [4]. Three H7N9 viruses were isolated—A/Anhui/1/2013, A/Shanghai/1/2013, and A/Shanghai/2/2013. A/Anhui/1/2013 and A/Shanghai/2/2013 were over 99% identical. In contrast, there were 53 nucleotide differences between A/Shanghai/1/2013 and A/Shanghai/2/2013 [5,6]. Since that time, sporadic human infections with Asian H7N9 viruses in China have been reported. H7N9 infection in humans is of concern because most patients become severely ill. So far, China has faced six epidemic waves of Asian H7N9 human infections. Because H7N9 viruses circulate continuously in domestic and wild birds and are also able to infect humans, they have been recognized as potentially pandemic and a global public health threat. Sustained human-to-human transmission of H7N9 viruses has not been reported. However, WHO has acknowledged the potentially serious impact of a human pandemic of H7N9 influenza. Since March 2013, the total number of human cases of H7N9 infection in China has reached 1568 including at least 615 deaths (39%) [7].

A number of candidates for inactivated whole virion or split vaccines, live, recombinant and DNA vaccines have been developed based on the A/Anhui/1/2013 [8,9,10,11,12,13,14,15,16] and A/Shanghai/2/2013 [17,18,19,20,21,22] antigenic prototype viruses. Clinical trials of these vaccine candidates were successfully completed between 2014 and 2017 [23,24]. Two more phase II clinical trials of inactivated H7N9 influenza vaccines from strains isolated in 2017 have been completed (ClinicalTrials.gov Identifiers NCT03312231 and NCT03318315, respectively), but information regarding the particular strains used for vaccine development is not yet available.

Currently circulating H7N9 viruses belong to two genetic hemagglutinin (HA) lineages—the Yangtze River Delta (YRD) and Pearl River Delta (PRD)—which are antigenically distinct from A/Anhui/1/2013-like viruses. Recent viruses of the YRD lineage reacted poorly with ferret antiserum derived from A/Anhui/1/2013 [25] and with human serum samples obtained in 2014–2015 from a phase I clinical trial of an A/Anhui/1/2013-based live attenuated influenza vaccine (LAIV) candidate [26].

In 2017, after analysis of H7N9 influenza activity, WHO proposed that new candidate vaccine viruses should be developed from A/Hunan/2650/2016-like (H7N9) strains of YRD HA lineage [25]. In response to this recommendation, in 2018 we developed an LAIV candidate against human A/Hong Kong/125/2017 strain of avian origin, a low-pathogenic A/Hunan/2650/2016-like virus, and studied its safety and immunogenicity in a ferret model [26]. This was the only live influenza vaccine based on the new YRD HA lineage virus to be included in the latest WHO tables on clinical evaluation of pandemic and potential pandemic influenza vaccines in August 2019 [24].

This study describes a double-blind randomized placebo-controlled phase I clinical trial in healthy adults of a new potential pandemic H7N9 Russian LAIV based on A/Hong Kong/125/2017 candidate.

## 2. Materials and Methods

### 2.1. Vaccine

The H7N9 LAIV candidate is a 6:2 gene substitution reassortant, constructed in the Institute of Experimental Medicine (St Petersburg, Russian Federation) by classical reassortment between the human A/Hong Kong/125/2017 (H7N9) wild type virus of avian origin (H7N9 WT) (an A/Hunan/2650/2016-like virus of YRD HA lineage) [25] and A/Leningrad/134/17/57 (H2N2), an attenuated cold-adapted master donor virus (L17 MDV) in embryonated chicken eggs [26]. In brief, a vaccine candidate between L17 MDV and H7N9 WT virus was produced in specific pathogen-free embryonated eggs (VALO BioMedia GmbH, Germany) following seven rounds of selective propagation. The production and selection for reassortants was undertaken in the presence of anti–MDV serum. Low temperature propagation (26 °C) was also used as a selective factor. The H7N9 LAIV candidate contains six gene segments encoding the internal proteins from the MDV, and the HA and neuraminidase (NA) genes from the WT virus. Both the H7N9 monovalent LAIV and the placebo (sterile phosphate buffered saline, PBS) were manufactured under Russian good manufacturing practice (GMP) regulations and supplied by Microgen (Irkutsk, Russian Federation). The formulation of the stabilizer and the LAIV production method are described elsewhere [27]. The vaccine was formulated to contain 7.0 log_10_ EID_50_ of H7N9 LAIV per 0.5 mL dose.

### 2.2. Study Design

The trial was a randomized, double-blind, placebo-controlled study to evaluate the safety, immunogenicity, and phenotypic and genotypic stability of H7N9 potential pandemic monovalent LAIV in healthy adults. Of 53 screened volunteers, 13 subjects (24.5%) did not meet the criteria for inclusion in the study and 40 (75.5%) eligible volunteers were randomly distributed into two groups to receive two doses of either LAIV or placebo, at a vaccine:placebo ratio of 3:1; thus, 30 subjects were assigned to the vaccine group and 10 to the placebo group. All 40 subjects received their assigned treatment on day 0, while 39 (97.5%) subjects received their assigned follow-up treatment on day 28 and completed the study as per protocol. The subject disposition flow diagram is presented in Figure S1. Vaccine and placebo recipients were not isolated from each other, two to four recipients being housed in one room. They were discharged from the isolation unit on day 6 after vaccination or revaccination.

### 2.3. Study Population

The study population comprised 40 healthy adult men and non-pregnant women aged 18–49 years who had received no vaccines in the previous 4 weeks and had not been included in another clinical trial in the previous 3 months.

### 2.4. Intervention

Vaccine and placebo were administrated intranasally (0.25 mL into each nostril) with a single-use nasal sprayer. Two doses were given at a 28-day interval.

### 2.5. Outcomes

The primary aim was to evaluate the safety profile of two intranasal doses of H7N9 LAIV in healthy adults. Secondary outcomes included evaluation of immunogenicity, and phenotypic and genotypic stability of H7N9 LAIV.

### 2.6. Trial Registration

ClinicalTrials.gov Identifier: NCT03739229.

### 2.7. Clinical Observation

For assessment of safety, subjects were observed for 2 hours after each administration of study vaccine or placebo, and for the following 6 days (early morning and late afternoon). The physical examination included the following: (i) recording of general appearance; (ii) physical examination of organ systems (e.g., neurological examination, chest auscultation); and (iii) measurement of weight, body temperature, blood pressure, heart rate and respiratory rate. An ear, nose and throat examination was carried out on days 6, 28, 34, and 56. Subjects completed diary cards for unsolicited adverse events from the day of first discharge (day 6) until return to the isolation unit for dose two (day 28) or until return to the study center for the final visit 4 weeks after dose two (day 56). Blood and urine specimens were collected on days 6, 28 (prior to administration of the second dose), 34, and 56; routine biochemical and hematological blood tests were carried out, as well as urinalysis by dipstick.

### 2.8. Ethical Approval

The study (Protocol Number: LAIV–H7N9–02) was approved by the Independent Ethics Committee under the Ministry of Health of the Russian Federation and the Research Ethics Committee, by the WHO Ethics Review Committee, and by the Local Ethics Committee of Smorodintsev Research Institute of Influenza, Ministry of Health of the Russian Federation, St Petersburg, Russian Federation. It was conducted in compliance with the Declaration of Helsinki. Written informed consent was obtained from each study participant (Supplementary Form S1).

### 2.9. Vaccine Virus Isolation in Chicken Eggs

Detection of LAIV virus shedding and recovery of viruses from nasal swabs obtained after vaccination (days 1–6 after each vaccine dose) was carried out by culture of 0.2 mL of clinical samples in 10–11-day-old chicken eggs (Siniavino Chicken Farm, Leningrad region, Russian Federation) followed by incubation at 32 °C for 72 h, in line with the WHO-recommended protocol [28]. Influenza virus was detected by standard hemagglutination test with 1% chicken red blood cells.

### 2.10. PCR-Based Vaccine Virus Detection

Nasal swabs for detection of LAIV virus shedding by real-time reverse transcriptase polymerase chain reaction (rRT–PCR) were obtained from each nostril prior to vaccine administration (day 0) and on days 1–6 after the first and the second vaccine dose, and mixed with transport medium. RNA was extracted from 100 µL of the nasal swabs using “RIBO-sorb” reagent kit (InterLabService, Moscow, Russia). Real-time PCR testing was performed using SuperScript III Platinum One-step qRT–PCR System (Invitrogen). Primers and probes to detect and subtype virus RNA were kindly provided by the US Centers for Disease Control (CDC, Atlanta, GA, USA).

### 2.11. Determining ts/ca Phenotype Stability of Clinical Isolates

The capacity of clinical isolates to grow at optimum (32 °C), low (26 °C) and elevated (40 °C) temperatures for *ts/ca* viruses was determined by titration in 10–11-day-old chicken eggs. The log_10_ EID_50_/mL calculation was based on the routine Reed and Muench method [29]. Viruses were considered as possessing *ts* phenotype if log_10_ EID_50_/mL at 32 °C – log_10_ EID_50_/mL at 40 °C ≥ 4.5 log_10_ EID_50_/mL. Viruses were considered as having a *ca* phenotype if log_10_ EID_50_/mL at 32 °C–log_10_ EID_50_/mL at 26 °C ≤ 3.0 log_10_ EID_50_/mL. Two influenza viruses were used for the quality control of phenotypic analysis: *non-ts/non-ca* A/Hong Kong/125/2017 (H7N9) wild-type virus (CDC ID #3000687670) and its *ts/ca* H7N9 LAIV candidate. All work with H7N9 influenza viruses was performed in a biosafety level 2 facility.

### 2.12. Determining Genotype Stability of Clinical Isolates

RNA was extracted from the chorioallantoic fluid of chicken eggs with QIAamp Viral RNA Mini Kit (QIAGEN, Hilden, Germany), according to the manufacturer’s protocol. PCR with reverse transcription was performed by Invitrogen™ Platinum™ SuperScript™ III One-Step RT–PCR System with Platinum Taq (Invitrogen, Carlsbad, CA, USA) with universal primers [30]. 

Partial sequencing of genome seqments of the clinical isolates was performed to confirm the 6:2 genome composition of isolates as described by Matyushenko [31]; the genetic stability of attenuating mutations in the internal genes was confirmed using the PyroMark Q24 (QIAGEN GmbH, Hilden, Germany). The results were analyzed using PyroMarkQ24 Software V 2.0.6. Samples were prepared for pyrosequencing in accordance with the protocol of the manufacturer of the PyroMarkQ24 instrument. Biotinylated primers were developed with PyroMark Assay Design software and synthesized by Evrogen (Moscow, Russia). For DNA strand separation, sepharose beads coated with streptavidin (Streptavidin Sepharose High Performance, GE Healthcare, Chicago, Illinois, USA) were used. DNA denaturation was then carried out, washing with a vacuum prep washing station, after which single-stranded DNA fragments immobilized on the sepharose beads were incubated in a buffer solution containing the primers of the sequencing step.

### 2.13. Hemagglutination Inhibition (HI) Test

Serum samples were pretreated with receptor-destroying enzyme (RDE, Denka-Seiken, Tokyo, Japan). Serum HI antibody titers were determined using standard HI assay adapted to identify HI antibody specific for H7N9 avian influenza viruses [32]. The HI assay was performed using 1.0% suspension of horse red blood cells and four hemagglutinating units (HAU) of vaccine antigen (H7N9 LAIV virus). Fourfold or greater increases in antibody titer after vaccination were considered as reliable increases (conversions).

### 2.14. Microneutralization (MN) Assay

MN assay was used to measure serum neutralizing antibody titers in sera using a Madin Darby canine kidney (MDCK) cell line with standard procedures [33]. H7N9 LAIV virus was used as an antigen. Fourfold or greater increases in antibody titer after vaccination were considered as reliable increases (conversions).

### 2.15. Measurement of Virus-Specific Antibodies

Virus-specific serum IgA and IgG antibodies were evaluated using enzyme-linked immunosorbent assay (ELISA) with 16 HAU of sucrose gradient purified vaccine antigen (H7N9 LAIV virus), since the purified HA protein of A/HK H7N9 virus was not commercially available at the time of the study. Antibody titer was determined as the last sample dilution that had optical density (OD) of more than double the mean of the control wells (all the reagents with PBS instead of sample). For serum IgG ELISA, the starting dilution was 1:100. For serum IgA ELISA, the starting dilution was 1:10. Fourfold or greater increases in antibody titer after vaccination were considered as reliable increases (conversions).

Nasal swabs were collected using ear packing with string (12 mm diameter × 15 mm long, Invotec, UK), which were then placed in a tube containing 0.5 mL of PBS, and centrifuged for 10 min at 3000 rpm. Because there was a high variability in the concentrations of total IgA antibody recovered from nasal wick specimens collected from the same patients at different times, the concentrations of virus-specific secretory IgA (sIgA) antibodies were measured by semi-quantitative ELISA, as described elsewhere [34]. Briefly, the concentrations of total IgA for each nasal swab specimen were measured in duplicates using a human IgA ELISA kit (Bender MedSystems GmbH, Vienna, Austria). For detection of antigen-specific IgA antibody, 96-well Microlon high-binding plates (Greiner bio-one, Frickenhausen, Germany) were coated with 0.2 µg per well of sucrose gradient-purified H7N9 LAIV virus overnight. After blocking and washing, diluted 1:4 nasal swab specimens were added, followed by incubation with goat anti-human IgA antibody conjugated with horseradish peroxidase (Sigma-Aldrich, St. Louis, MO, USA). The chromogen was produced using BD OptEIA™ TMB Substrate Reagent Set (Becton Dickinson, Franklin Lakes, NJ, USA) and measured at an absorbance of 450 nm. Since there is no commercially available standard for HK/H7N9-specific human IgA antibody, some wells in the same ELISA plates were coated with rabbit anti-human IgA, α-chain-specific polyclonal antibody (Jackson ImmunoResearch, Ely, UK) and incubated with a series of dilutions of a standard human IgA antibody (Thermo Fisher Scientific, USA). The levels of virus-specific sIgA antibody in the swabs were expressed in IgA units (U) from the regression curve of the standard IgA titration, where one unit corresponded to 1 µg/mL of the standard. The relative values of anti-H7N9 sIgA antibody were then normalized to the total concentration of IgA antibody in the sample and the virus-specific sIgA concentrations were expressed as:

$$\mathrm{Normalized}\;\mathrm{virus}-\mathrm{specific}\;\mathrm{sIgA}=\frac{\mathrm{virus}\text{-}\mathrm{specific}\;\mathrm{sIgA}\;\left(\frac{U}{\mathrm{mL}}\right)}{\mathrm{total}\;\mathrm{IgA}\;\left(\frac{\text{µ}g}{\mathrm{mL}}\right)}\times 100$$

Twofold or higher increases in the normalized concentrations of virus-specific sIgA antibody after vaccination were considered significant and treated as mucosal antibody responses. 

### 2.16. T-Cell Immune Responses

Virus-specific CD4+ and CD8+ T lymphocytes were measured by flow cytometry. Peripheral blood mononuclear cells were isolated using Leucosep Centrifuge Tubes (Grainer bio-one, Frickenhausen, Germany), washed and stored in liquid nitrogen until analysis. The frozen cells were thawed and washed, and then 2 × 10^6^ cells were stimulated in 96-well plates with purified vaccine strain at a multiplicity of infection (MOI) 5. To determine spontaneous cytokine production, an appropriate volume of RPMI-1640 nutrient medium was added to the cells instead of the stimulator. In the analysis, the results for these negative controls were subtracted from the values obtained for the virus-stimulated cells. Cell stimulation by phorbol myristate acetate (PMA) and ionomycin (both from Sigma-Aldrich, St. Louis, MO, USA), which induced abundant nonspecific activation of T lymphocytes, was used as a positive control. After thawing, cells collected from all volunteers at all time points were capable of activation and cytokine production. The fluorescently labeled antibody panel (all from Biolegend, San Diego, CA, USA) was used to identify CD3 (Clone: SK7), CD4 (Clone: SK3), CD8 (Clone: HIT8a), CD45RA (Clone: HI100), and CCR7 (also known as CD197) (Clone: G043H7) surface markers, which allow the detection of subsets of human T cells, such as central memory T cells (Tcm, CD45RA−CCR7+), effector memory T cells (Tem, CD45RA−CCR7−) and effector memory re-expressing CD45RA T cells (TEMRA, CD45RA + CCR7−) [35,36]. Dead cells were identified using Zombie Aqua (Biolegend, San Diego, CA, USA) viability dye. The T-cell response was analyzed by determining the relative content of cytokine-secreting cells. IFNγ (Clone: 4S.P3, Biolegend, San Diego, CA, USA) antibody and the BD Cytofix/Cytoperm™ Fixation/Permeabilization Solution Kit (Becton Dickinson, Franklin Lakes, NJ, USA) were used for intracellular cytokine staining (ICS). The data were collected using a Cytoflex flow cytometer (Beckman Coulter, Brea, CA, USA) and analyzed with Kaluza Analysis v2.1 and RStudio software, R version 3.6.0. Gating strategy is presented on Supplementary Figure S2.

### 2.17. Statistical Analysis

Statistical analysis of the data was performed with GraphPad Prizm 5 software using the following methods: Wilcoxon Matched Pairs Test (exact *p*), Mann–Whitney U test (two-sided exact *p*) and two-way ANOVA followed by a Bonferroni multiple comparison tests.

## 3. Results

### 3.1. Study Population Characteristics

Baseline indicators and descriptive statistics for the key demographic and other characteristics are summarized in Table 1. Of the 40 subjects enrolled in the study, 24 (60.0%) were male and 16 (40.0%) were female. The distribution of males and females in the two treatment arms was well balanced; the median age of subjects was 33.0 years in the vaccine group and 31.0 years in the placebo group. Median baseline height was similar in the two groups: 173.5 cm in the vaccine group and 174.5 cm in the placebo group. Median baseline weight was lower in the vaccine group (65.0 kg) than in the placebo group (74.0 kg). All subjects had normal physical examination findings at study entry. No subjects had a history of allergic reactions.

### 3.2. Reactogenicity

All subjects were monitored closely for local and systemic reactions and adverse events (AEs) for 7 days following each dose of study vaccine or placebo. A summary of the adverse reactions and adverse events that occurred is given in Table 2. No immediate reactions were reported in the first 2 h after vaccination with either the first or the second dose. During the 6 days after administration of the first dose, three subjects in the vaccine group (10.0%) and none in the placebo group reported solicited local and systemic AEs. One vaccine recipient (3.3%) reported a local AE (nasal congestion). Systemic AEs included fatigue, malaise and fever. During the 6 days after administration of the second dose, one (3.3%) local AE (nasal congestion) was observed in the vaccine group and none in the placebo group. Five systemic adverse events were recorded in the placebo group and none in the vaccine group. All registered AEs were evaluated as mild and self-limiting.

A detailed overview of the solicited local and systemic adverse reactions and adverse events is given in Supplementary Table S1. Supplementary Table S2 summarizes the laboratory abnormalities found in the 6 days after each dose. Clinical laboratory evaluations showed no significant abnormalities and were deemed to be of no clinical significance by the study physicians.

There were no immediate reactions to vaccination and no serious adverse events during this study. Recorded AEs during the six days after vaccination were infrequent, and consisted of expected events that were mild and self-limiting. Clinical laboratory evaluations showed no significant abnormalities and were deemed to be of no clinical significance by the study physicians.

Overall, LAIV H7N9 vaccine administered in two intranasal doses was found to be well tolerated and safe.

### 3.3. Vaccine Virus Shedding

#### 3.3.1. Vaccine Virus Isolation in Embryonated Chicken Eggs

Infectious vaccine virus was isolated by culturing nasal swabs in chicken eggs. Nineteen H7N9 vaccine virus samples were isolated after dose 1 and six samples were recovered after dose 2, bringing the total to 25 (Table 3).

Only one subject shed the vaccine virus after both vaccination and revaccination. In total, 12 out of 30 vaccinees (40.0%) shed H7N9 vaccine virus after the first or the second dose (Supplementary Table S3).

#### 3.3.2. Vaccine Virus Detection by RT-PCR

All subjects were monitored for the presence of vaccine virus in nasal swabs by rRT–PCR 2 h before vaccination, on days 1–6 after the first vaccination and on days 28–34 (days 1–6 after the second vaccination). Influenza virus H7N9-specific sequences were found in 27 of the 30 vaccinees on day 1; 8 on day 2; 5 on day 3; 3 on day 4; 1 on day 5; and 1 on day 6 after the first vaccination (Table 3). After the second vaccination, H7N9 viral RNA was found in 9 of the 30 vaccinees on day 1, 4 on day 2, 1 on day 3 and 2 on day 4. In total, 28 of the 30 vaccinees (93.3%) shed H7N9 vaccine virus after the first or the second dose of vaccine (Supplementary Table S3). 

### 3.4. Vaccine Virus trAnsmission to Placebo Recipients

Placebo recipients were also tested for the presence of vaccine virus in nasal swabs during the post-vaccination period by RT–PCR and culturing in chicken eggs. No vaccine virus was recovered after three sequential passages in chicken eggs, and RT–PCR did not detect vaccine virus in nasal swabs (Table 3). Thus, although vaccinees and placebo recipients were in close contact, there was no confirmed transmission of the H7N9 vaccine virus between them. 

### 3.5. Vaccine Virus Stability after Replication in Humans

The nasal swab isolates were tested for the stability of the 6:2 vaccine genotype, for the presence of all attenuating mutations in internal genes, and for the attenuated *ts/ca* phenotype.

#### 3.5.1. Confirmation of 6:2 Genotype and Genetic Stability 

The 6:2 vaccine reassortant genotype was confirmed for all 25 clinical isolates by pyrosequencing. In addition, the presence of all attenuating mutations known for L17 MDV (*n* = 8) was confirmed (Supplementary Table S4).

The 25 viral isolates were tested for the presence of amino acid changes in surface proteins, HA and NA. HA and NA genes were full-length sequenced. No additional nucleotide and amino acid changes were detected in any H7N9 LAIV isolate (data not shown), suggesting that the vaccine virus surface antigens are highly genetically stable.

#### 3.5.2. Phenotypic Stability of Vaccine Virus Clinical Isolates

All viral isolates were investigated for their ability to reproduce at temperatures above and below the optimum, to confirm their *ts* and *ca* phenotype specific for LAIV on the backbone of L17 MDV.

**Figure 1:**
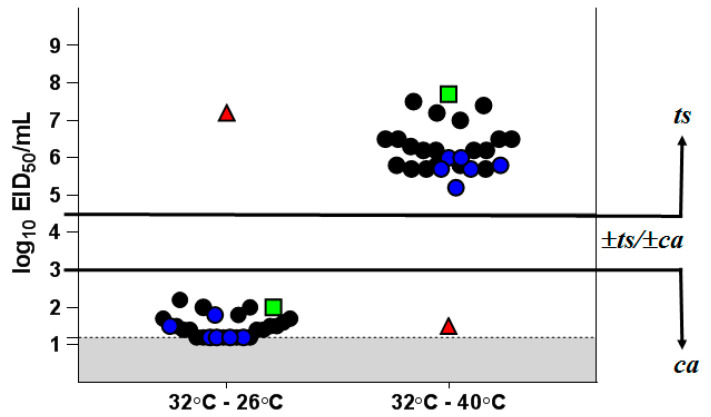
Growth restriction of H7N9 LAIV clinical isolates at different temperatures. The results are expressed as the reduction in virus titer at 40 °C or 26 °C from the titer at the optimum temperature of 32 °C. Black circles—isolates obtained after the first vaccine dose; blue circles—isolates obtained after the second vaccine dose; red triangle—H7N9 WT virus; green square—H7N9 LAIV candidate; gray—the limit of virus detection (1.2 log_10_ EID_50_/mL).

As shown in Figure 1, H7N9 LAIV itself and its clinical isolates had a clear *ts/ca* phenotype. There was an almost complete lack of reproduction at 40 °C (virus titer at 40 °C was about 5–8 log_10_ EID_50_/mL lower than that at 32 °C), while at 26 °C the titer was only 1.3–2.0 log_10_ EID_50_/mL below that at 32 °C. In contrast, parental wild-type influenza virus A/Hong Kong/125/2017 (H7N9) possesses a *non-ts* and *non-ca* phenotype. It multiplies at 40 °C as well as at the optimum temperature of 32 °C and is almost unable to reproduce at 26 °C (Supplementary Table S5). These results confirm the stability of the *ts/ca* phenotype of the isolates from vaccinated individuals.

### 3.6. Immune Responses

**Figure 2:**
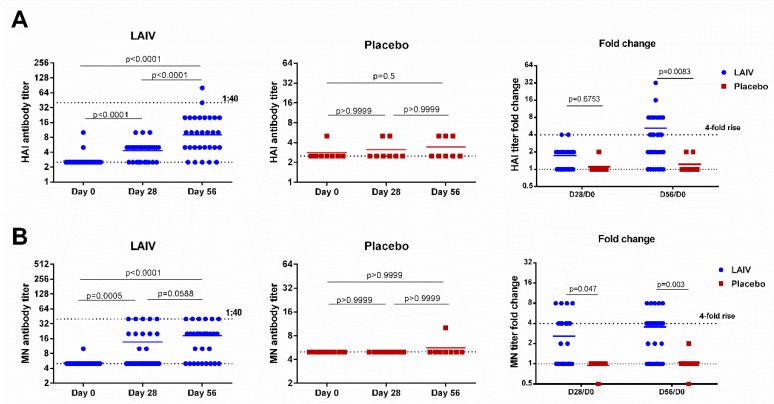
Hemagglutination inhibition and neutralizing antibody titers in subjects immunized with H7N9 LAIV or placebo. (**A**) HAI antibody titers. (**B**) MN antibody titers. Data were analyzed by Wilcoxon Matched Pairs Test (left and middle panels) and two-way ANOVA followed by a Bonferroni multiple comparison test (right panel).

H7N9 LAIV was immunogenic for healthy adults: The virus-specific HAI and MN antibody titers after the first dose were significantly higher than the pre-vaccination levels, and were further boosted by the second vaccination (Figure 2). There were no increases in HAI and MN antibody titers in placebo recipients, and the fold rises in antibody levels were significantly different in the two test groups on day 56 for both assays. The MN assay showed a statistically significant difference already on day 28 (*p* = 0.047, Figure 2). Despite these significant increases, the antibody titers remained relatively low, with only two (6.7%) and six subjects (20%) reaching a titer of 1:40 in HAI and MN assays, respectively. 

**Figure 3:**
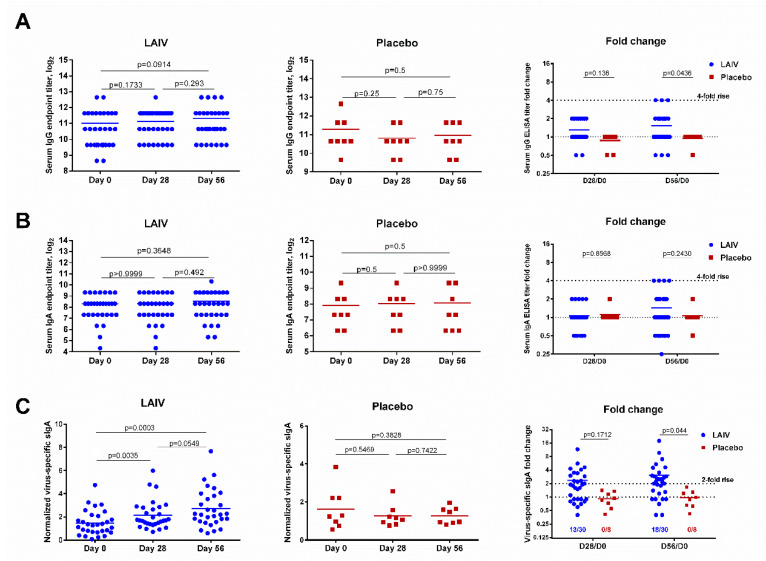
Virus-specific serum IgG and IgA antibody titers and nasal secretory IgA antibody levels in subjects immunized with H7N9 LAIV or placebo. (**A**) Serum IgG titers. (**B**) Serum IgA titers. (**C**) Nasal secretory IgA antibody levels. Data were analyzed by Wilcoxon Matched Pairs Test (left and middle panels) and two-way ANOVA followed by a Bonferroni multiple comparison test (right panel).

Unlike the HAI and MN assay results, virus-specific IgG and IgA antibody levels were not significantly increased after one or two doses of H7N9 LAIV, and only three (10%) and four (13.3%) subjects seroconverted by IgG and IgA antibody, respectively (Figure 3). Nevertheless, serum IgG antibody fold change values were significantly higher in the LAIV group than the placebo group on day 56 of the study (*p* = 0.0436, Figure 3). 

Concentrations of virus-specific sIgA significantly increased in immunized subjects after the first LAIV dose (*p* = 0.0035, Figure 3C). The second LAIV dose further boosted these antibody levels. Although the difference between antibody levels on days 28 and 56 was not statistically significant (*p* = 0.0549), the fold increase for LAIV recipients at day 56 was significantly higher than that for placebo recipients (*p* = 0.044, Figure 3C). Unlike serum antibody titers, which are determined using twofold serial dilutions (with seroconversion usually defined as a fourfold increase in titer), the virus-specific sIgA concentration is a continuous parameter. A twofold rise in the normalized virus-specific sIgA levels post-vaccination was therefore defined as a significant increase. Using this threshold, significant virus-specific sIgA antibody increases were observed in 13 (43.3%) vaccinated subjects after the first LAIV dose and 18 (60%) after the second dose (Figure 3C). As expected, no significant rise in sIgA was seen in the placebo recipients. 

Supplementary Table S6 summarizes the data on serum and secretory antibody responses after two doses of H7N9 LAIV. After the first dose, 23 vaccinated subjects (76.7%) responded according to at least one of the antibody assays performed. The majority of antibody rises after a single LAIV dose were registered in the microneutralization test and nasal sIgA ELISA (33.3% and 43.3% responses, respectively). After the second vaccination, the number of antibody conversions detected by the various assays increased to 10.0–60.0%. The largest number of conversions was registered by HAI, MN and nasal sIgA ELISA assays (53.3%, 60% and 60%, respectively). The lowest number of conversions was noted when measuring serum IgG and IgA antibody by ELISA (10.0% and 13.3%, respectively). Overall, after the second LAIV dose, 29 (96.7%) vaccinated subjects responded in at least one immunological assay, whereas no antibody conversions were observed in the placebo group (Supplementary Table S6).

**Figure 4:**
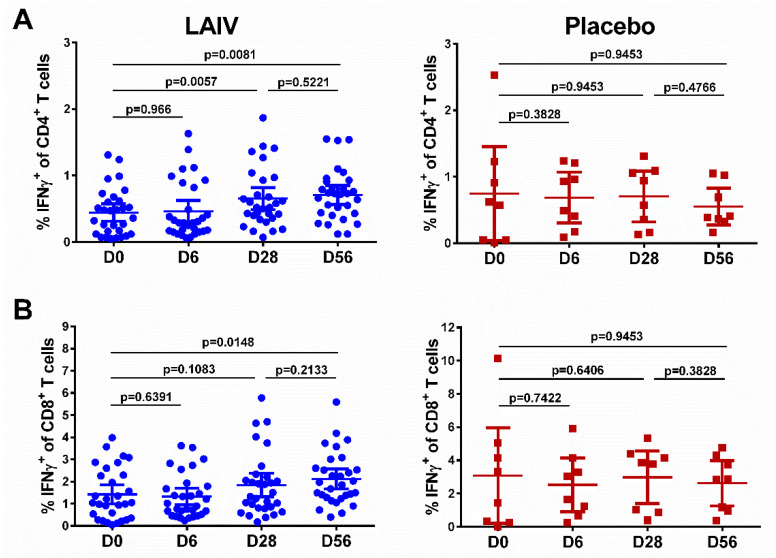
Cell-mediated immune responses in subjects before and after administration of H7N9 LAIV or placebo (given on day 0 and day 28). (**A**) CD4^+^ T-cell responses. (**B**) CD8^+^ T-cell responses. Individual data are shown, along with means ± 95% CI. T cell levels at different time-points were compared using the Wilcoxon matched-pairs test. The exact two-sided *p* values are shown.

The baseline levels of IFNγ-producing T cells varied significantly among study participants, probably reflecting the population of T cells specific to cross-reactive influenza virus epitopes, since we used whole virus antigen (Figure 4, day 0). The total percentage of cytokine-positive CD4^+^ lymphocytes increased after the first vaccination in the LAIV group (*p* = 0.0057, Figure 4A). The second LAIV dose did not further boost this T-cell subset, although a statistically significant difference remained in the LAIV group between day 0 (the baseline level) and day 56 (*p* = 0.0081). In contrast to the CD4^+^ T cells, two H7N9 LAIV doses were required to significantly increase virus-specific CD8^+^ T-cell levels in peripheral blood (*p* = 0.0148, Figure 4B). No statistically significant changes in the levels of CD4^+^ or CD8^+^ virus-specific T cells were detected in the placebo group at any time (Figure 4).

**Figure 5:**
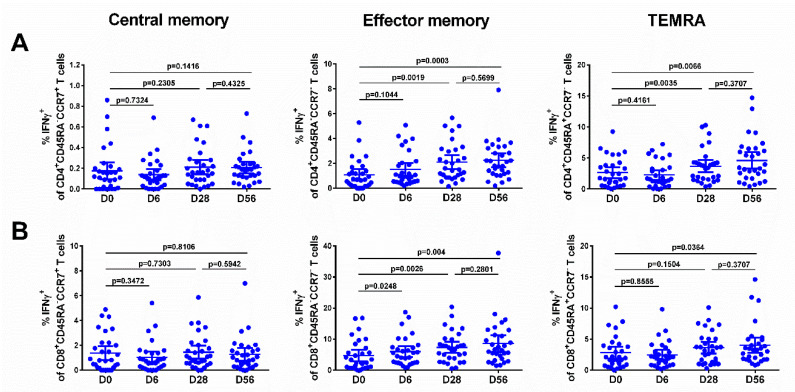
Different populations of IFNγ-producing CD4^+^ (**A**) and CD8^+^ (**B**) T cells in subjects immunized with H7N9 LAIV. Individual data are shown, along with means ± 95% CI. T cell levels at different time-points were compared using the Wilcoxon matched-pairs test.

**Figure 6:**
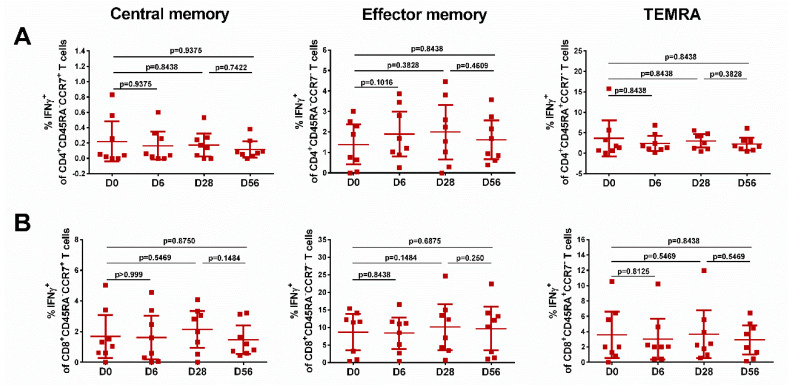
Different populations of cytokine-producing CD4 (**A**) and CD8 (**B**) T cells in subjects who received placebo. Individual data are shown, along with means ± 95% CI. T cell levels at different time-points were compared using the Wilcoxon matched-pairs test.

The levels of effector memory T cells, but not central memory T cells, increased after vaccination, and were significantly higher than the baseline after the first and the second dose, for both CD4^+^ and CD8^+^ subsets (Figure 5, middle panel), although no booster effect of the second LAIV dose was noted. Interestingly, significant levels of CD8+ virus-specific Tem cells had been induced by day 6 after the first LAIV dose (*p* = 0.0248, Figure 5B); this was the only T-cell subset that was significantly increased at this time. The frequency of IFNγ-secreting CD4^+^ TEMRA cells was also increased on day 28 after the first LAIV dose, whereas two doses were required to produce a significant increase of the CD8^+^ TEMRA cells. No significant increases in any of the T-cell subsets were seen in the placebo group (Figure 6).

## 4. Discussion

In contrast to other respiratory viruses, type A influenza viruses display high genetic flexibility. They circulate in different species of mammals and birds [37], undergo frequent genetic reassortment and have a high mutation rate that leads to variability in the surface proteins, HA and NA [38]. This natural genetic variability of influenza A virus means that recommendations on the composition of seasonal influenza virus vaccines need to be updated twice a year, for the northern and the southern hemisphere [39]. 

No less difficult is a situation with potentially pandemic influenza A viruses. It is not possible to predict which virus might become pandemic or when. Therefore, the development of vaccines against potentially pandemic influenza viruses is a key part of the WHO global influenza pandemic preparedness plan [40]. 

Clinical infections with avian influenza viruses are reported sporadically in humans. Some subtypes can cause mild, severe and even fatal disease, e.g., H5N6 [41,42,43], H7N7 [44], H5N1 [45], and H9N2 [46,47]. Recently, H7N9 avian influenza viruses have been reported most often in clinical cases [48,49]; most clinical cases caused by H7N9 viruses have been serious.

The situation is no less stark than it was 22 years ago when the first fatal case of human infection with avian influenza virus was reported [45,50]. Currently, WHO makes recommendations regarding the composition of not only seasonal influenza vaccines, but also potential pandemic vaccines [51].

Currently, three types of influenza vaccines—inactivated, live attenuated and recombinant—are available on the market. In recent years, the interest in LAIV has increased, largely because WHO has recognized a number of advantages of LAIV in the event of a pandemic: needle-free administration; protection against drifted variants; high vaccine virus yield (estimated to be at least ten times higher than for inactivated vaccines); easy downstream processing, etc. [52].

Under the WHO Global Influenza Pandemic Preparedness Plan [40], five live candidate vaccines have been developed on an L17 MDV backbone and tested in human trials [11,53,54,55,56]. The first Russian LAIV, based on A/duck/Potsdam/1402–6/86 (H5N2) virus, was registered in 2008 [54]. Further development included the production of potential pandemic LAIVs on an L17 MDV backbone against A/mallard/Netherlands/12/2000 (H7N3) [55], A/turkey/Turkey/1/05 (H5N1) [56], A/California/1/66 (H2N2) [53], and A/Anhui/1/2013 (H7N9) [11] viruses (Table 4). 

Seasonal LAIVs are formulated to contain 6.5–7.0 log_10_ EID_50_ vaccine virus per 0.5 mL dose [24]. The majority of potential pandemic LAIVs contain vaccine virus in a dose of 6.5–7.5 log_10_ TCID_50_ [24]. All the potential pandemic vaccines presented in Table 4 were formulated to contain 6.9–7.5 log_10_ EID_50_ vaccine virus per 0.5 mL dose, except the A/17/turkey/Turkey/05/133 (H5N2)-based vaccine, which contained 8.4 log_10_ EID_50_ vaccine virus per dose.

Since the emergence of H7N9 influenza viruses in 2013, a number of safe and immunogenic vaccine candidates have been evaluated in clinical trials, most of which were based on the A/Anhui/1/2013 strain [23]. In 2017, YRD and PRD genetic HA lineages, which are antigenically distinct from the previously dominant A/Anhui/1/2013-like viruses, appeared in circulation. WHO recommended the development of new candidate vaccine viruses from A/Hunan/2650/2016-like (H7N9) strains of the most promising YRD HA lineage [25]. A number of new H7N9 inactivated vaccine candidates have since been developed [24]. No information is available on H7N9 LAIV candidates yet.

Our study was a first double-blind randomized placebo-controlled phase I clinical trial of a new potential pandemic H7N9 LAIV candidate, representing a reassortant of A/Hunan/2650/2016-like A/Hong Kong/125/2017 WT virus with L17 MDV. In this clinical trial, the A/17/Hong Kong/2017/75108 (H7N9) LAIV elicited only mild adverse events, none of which are regulated by the Russian requirements for LAIV. In particular, after the first vaccination, 3.3% (1/30) of participants displayed mild local reactions, and 13.3% (4/30) systemic reactions. After the second dose of vaccine, only 3.3% (1/30) had local reactions while none had systemic reactions. No serious adverse events were detected after either dose 1 or dose 2.

A/17/Hong Kong/2017/75108 (H7N9) LAIV demonstrated a moderate level of vaccine virus replication in humans (40%), similar to that shown by A/17/California/66/395 (H2N2) (50.0%) [53] and A/17/turkey/Turkey/05/133 (H5N2) (46.7%) [56]. In contrast, 73.3% of vaccinees given A/17/Anhui/2013/61 (H7N9) LAIV [11] and only 13.3% of vaccinees given A/17/mallard/Netherlands/90/95 (H7N3) LAIV [55] shed vaccine virus (Table 4). 

Phenotypic (cold adaptation and temperature sensitivity) and genotypic analyses conducted on the viruses recovered from the study subjects suggest that the vaccine is genetically stable after in vivo passage. These data confirmed our previous findings that not only seasonal, but also pandemic and potential pandemic LAIVs on an L17 MDV backbone are genetically and phenotypically stable after replication in humans [58,59,60,61].

Many published studies have shown that seasonal LAIVs on an L17 MDV or A/Ann Arbor/6/60 MDV backbone are not transmitted from vaccinated adults and children to their unvaccinated close contacts [62,63]. There has been only one observation of transmission of the B component of a seasonal LAIV on the A/Ann Arbor/6/60 MDV backbone to a child in the placebo group, confirmed by virus isolation in MDCK cells [64]. Numerous clinical trials have demonstrated the absence of transmission of potentially pandemic LAIVs derived from L17 MDV [11,53,55,56]. In the current study, transmission from H7N9 LAIV recipients to non-vaccinated contacts was not detected by either PCR or nasal swab culture in chicken eggs. This supports the finding of a high level of safety and stability of LAIV based on L17 MDV.

The above-mentioned differences in the level of vaccine virus replication in volunteers, as well as differences in the concentration of the vaccine virus in each 0.5 mL dose, did not greatly influence the cumulative immune response: all but one immunized subject responded in at least one of the antibody assays (Supplementary Table S6). However, despite the conversions in virus-specific serum antibody, titers remained relatively low after vaccination, similar to the H2N2, H5N2, and H7N3 pandemic LAIVs previously tested in healthy adults [53,55,56], but were considerably lower than those induced by A/Anhui/1/2013-based LAIV. The latter vaccine is notably more immunogenic, since the majority of subjects achieve a seroprotective HAI and MN antibody titer after the second vaccine dose [11]. In line with previous pandemic LAIV trials, ELISA was a less-sensitive assay for detecting H7N9 virus-specific antibody, probably as a result of the use of whole virus as a coating antigen; this could bind pre-existing antibodies against the influenza virus NP and M1, since relatively high serum IgG and IgA antibody titers were seen at a baseline (Figure 3). The limitation of our study is that we did not measure the levels of HA stalk-specific antibodies post-LAIV. It is known that a recall response of stalk-specific memory B cells generated by previous exposure to H3N2 influenza viruses (both H3 and H7 belong to a group 2 HA) can be induced after vaccination with H7N9 vaccines [65].

It should be noted that, unlike in previous pandemic LAIV trials, mucosal IgA antibody levels in this study were assessed as virus-specific sIgA units normalized to total IgA concentration, to ensure that IgA levels were not dependent on the nasal wick sample collection efficiency [34]. This modified ELISA allowed significant rises in sIgA antibody concentration to be detected after H7N9 LAIV receipt, which were not seen in the standard ELISA (data not shown). This indicates that normalization to the total IgA concentration is critical for the assessment of immunogenicity of mucosal influenza vaccines.

It is widely accepted that vaccines capable of eliciting cell-mediated immune responses have many advantages over the currently used influenza vaccines, because of the cross-reactive nature of cellular immunity [66,67]. In addition, the primary goal of cell-mediated viral vaccines is the generation of protective T-cell memory, which is maintained across the lifespan of the individual and provides substantial protection against subsequent viral infections [68]. Importantly, the new H7N9 LAIV had similar immunogenicity to the earlier H7N9 LAIV in terms of induction of virus-specific CD4^+^ and CD8^+^ T-cell responses. Both vaccines elicited substantial levels of IFNγ-secreting CD4^+^ T cells after a single vaccination, while two LAIV doses were required to produce significant increases in the levels of CD8^+^ T cells [11]. Further assessment of the memory T-cell subsets found significant increases in Tem and TEMRA (but not Tcm) populations after H7N9 LAIV receipt, suggesting that the induced cellular immunity is durable. 

## 5. Conclusions

Overall, this phase I clinical trial in healthy adults showed the new H7N9 LAIV to be safe and well tolerated. It induced significant antibody- and cell-mediated immune responses and has the potential to be used as the first line of defense in the event of an influenza pandemic that is caused by the H7N9 virus.

## Figures and Tables

**Table 1:** Baseline characteristics of the study population.

Characteristics		Group	
		Vaccine, *n* (%)	Placebo, *n* (%)
Gender	Male	18/30 (60.0)	6/10 (60.0)
	Female	12/30 (40.0)	4/10 (40.0)
Age (years)	Mean (SE ^1^)	32.6 (9.8)	34.8 (9.3)
	Median	33.0	31.0
	Range	18.0–48.0	25.0–49.0
Weight (kg)	Mean (SE)	68.1 (11.5)	74.6 (3.8)
	Median	65.0	74.0
	Range	50.0–94.0	70.0–81.0
Height (cm)	Mean (SE)	175.6 (10.1)	175.1 (7.0)
	Median	173.5	174.5
	Range	160.0–210.0	166.0–185.0

^1^ SE = Standard error.

**Table 2:** Adverse reactions in the 6 days after vaccination or revaccination.

Adverse Reaction	Worst Grade ^2^	Group			
		Vaccine (*n* = 30)		Placebo (*n* = 8)	
		N ^3^ (%)	95% CI	N ^3^ (%)	95% CI
**Dose 1**					
Reactions within 2 h	none	0/30 (0)		0/8 (0)	
Any solicited local or systemic reaction ^1^		3/30 (10.0)	3.5–25.6	0/8 (0)	0.0–32.4
Local reactions (total)		1/30 (3.3)	0.6–16.7	0/8 (0)	0.0–32.4
Nasal congestion	mild	1/30 (3.3)	0.6–16.7	0/8 (0)	0.0–32.4
Systemic reactions (total)		4/30 (13.3)	5.3–29.7	0/8 (0)	0.0–32.4
Fatigue	mild	1/30 (3.3)	0.6–16.7	0/8 (0)	0.0–32.4
Malaise	mild	1/30 (3.3)	0.6–16.7	0/8 (0)	0.0–32.4
Fever	mild	2/30 (6.7)	1.8–21.3	0/8 (0)	0.0–32.4
Worst grade (total)		mild (*n* = 5)		None	
Any serious adverse event		none		None	
**Dose 2**					
Reactions within 2 h	none	0/30 (0)		0/8 (0)	
Any solicited local or systemic reaction ^1^		1/30 (3.3)	0.6–16.7	3/8 (37.5)	13.7–69.4
Local reactions (total)		1/30 (3.3)	0.6–16.7	0/8 (0)	0.0–32.4
Nasal congestion	mild	1/30 (3.3)	0.6–16.7	0/8 (0)	0.0–32.4
Systemic reactions (total)		0/30 (0)	0.0–11.4	5/8 (62.5)	30.6–86.3
Cough	mild	0/30 (0)	0.0–11.4	1/8 (12.5)	2.2–47.1
Sore throat	mild	0/30 (0)	0.0–11.4	1/8 (12.5)	2.2–47.1
Fever	mild	0/30 (0)	0.0–11.4	1/8 (12.5)	2.2–47.1
Herpes	mild	0/30 (0)	0.0–11.4	1/8 (12.5)	2.2–47.1
Hyperemia arches throat	mild	0/30 (0)	0.0–11.4	1/8 (12.5)	2.2–47.1
Worst grade (total)		mild (*n* = 1)		mild (*n* = 5)	
Any serious adverse event		none		None	

^1^ A subject with more than one reaction in a specific category was counted only once. ^2^ Severity was quantified as follows: none—lack of sign or symptom, normal; mild—events require minimal or no treatment and do not interfere with the subject’s daily activities; moderate—events result in a low level of inconvenience or concern to the subject with therapeutic measures; severe—events interrupt the subject’s functioning and might require systemic drug therapy or other treatment. ^3^ Percentages based on total number of subjects in each treatment group.

**Table 3:** Detection of influenza H7N9 LAIV virus in nasal swabs in the 6 days after vaccination or revaccination.

Vaccination	Group ^1^	Virus Isolation Confirmed by	Clinical Isolates						Total No. of Positive Subjects	
			D1	D2	D3	D4	D5	D6	Total No. of Isolates	
Dose 1	Vaccine (*n* = 30)	RT–PCR	27/30	8/30	5/30	3/30	1/30	1/30	45	27/30 (90.0%)
		Culture	6/30	5/30	5/30	3/30	0/10	0/10	19	11/30 (36.7%)
	Placebo (*n* = 8)	RT–PCR	0/8	0/8	0/8	0/8	0/8	0/8	0	0/8 (0%)
		Culture	0/8	0/8	0/8	0/8	0/8	0/8	0	0/8 (0%)
Dose 2	Vaccine (*n* = 30)	RT–PCR	9/30	4/30	1/30	2/30	0/30	ND ^2^	16	4/30 (13.3%)
		Culture	1/30	2/30	1/30	2/30	ND	ND	6	3/30 (10.0%)
	Placebo (*n* = 8)	RT–PCR	0/8	0/8	0/8	0/8	ND	ND	0	0/8 (0%)
		Culture	0/8	0/8	0/8	0/8	ND	ND	0	0/8 (0%)

^1^ All subjects were negative before vaccination. ^2^ ND—not determined.

**Table 4:** Vaccine virus shedding and immunogenicity of potential pandemic LAIVs on L17 MDV backbone in healthy adults.

LAIV Based on Vaccine Candidate [ClinicalTrials.Gov Identifier]	Dose Per 0.5 mL	Vaccine Virus Shedding after 1 and 2 Doses, Total Number		Immunogenicity (Any Test) ^3^	Reference
		Isolates ^1^	Subjects ^2^		
A/17/duck/Potsdam/88/92 (H5N2) [No data]	6.9 log_10_ EID_50_	25	No data	80.0% (16/20)	[57]
A/17/mallard/Netherlands/90/95 (H7N3) [NCT01511419]	7.0 log_10_ EID_50_	4	4 (13.3%)	86.2% (25/29)	[55]
A/17/turkey/Turkey/05/133 (H5N2) [NCT01719783]	8.4 log_10_ EID_50_	16	14 (46.7%)	96.6% (28/29)	[56]
A/17/California/66/395 H2N2) [NCT01982331]	7.5 log_10_ EID_50_	20	14 (50.0%)	92.6% (24/26)	[53]
A/17/Anhui/2013/61 (H7N9) [NCT02480101]	7.5 log_10_ EID_50_	45	22 (73.3%)	93.1% (27/29)	[11]
A/17/Hong Kong/2017/75108 (H7N9) [NCT03739229]	7.0 log_10_ EID_50_	25	12 (40.0%)	96.6% (29/30)	Current study

^1^ Infectious vaccine viruses isolated by culturing nasal swabs in chicken eggs. ^2^ Subjects who shed vaccine virus. ^3^ Subjects who developed an immune response (*n*, %) as measured by any assay.

## Supplementary Materials

The following are available online at https://www.mdpi.com/2076-393X/8/2/296/s1. Table S1: Solicited local and systemic adverse reactions in the six days after vaccination or revaccination. Table S2: Laboratory abnormalities in the six days after vaccination or revaccination. Table S3: Results of RT–PCR testing of volunteers’ nasal swabs after immunization with A/17/Hong Kong/2017/75108 (H7N9) LAIV and detection of vaccine virus by culturing nasal swabs in embryonated chicken eggs. Table S4: Stability of attenuating mutations in internal genes of H7N9 LAIV clinical isolates. Table S5: Reproductive activity of the A/17/Hong Kong/2017/75108 (H7N9) LAIV clinical isolates in developing chicken embryos at various incubation temperatures. Table S6: Antibody increases in volunteers after vaccination with H7N9 LAIV or placebo, as measured in different assays. Figure S1: Subject disposition flow diagram. Figure S2: Gating strategy for analysis of virus-specific T-cell populations in peripheral blood of subjects immunized with H7N9 LAIV or placebo. Form S1: Subject information sheet and participants’ inform consent form *B*.
