# Supplementary material for: A Phase 1 Randomized Placebo-Controlled Study to Assess the Safety, Immunogenicity and Genetic Stability of a New Potential Pandemic H7N9 Live Attenuated Influenza Vaccine in Healthy Adults
Source: Vaccines (Basel). 2020 Jun 10;8(2):296. doi: 10.3390/vaccines8020296 (PMC7350028; doi:10.3390/vaccines8020296)
Supplement: Supplementary file 1 [file vaccines-08-00296-s001.zip › Supplementary Table S1.pdf]

**Table S1.** Solicited local and systemic adverse reactions in the six days after vaccination or revaccination

| Group | Volunteer ID | Adverse reactions | Day of the study |
|---|---|---|---|
| **Adverse reactions after dose 1** | | | |
| Vaccine | 1 | Body temp 37.0 °C | Day 2 (evening) – Day 3 (morning) |
| | | Malaise | Day 2 (evening) – Day 3 (morning) |
| | | Fatigue | Day 2 (evening) – Day 3 (morning) |
| Vaccine | 2 | Body temp 37.1 °C | Day 2 – Day 4 |
| | | Body temp 37.2 °C | Day 4 – Day 6 |
| | | Nasal congestion | Day 4 – Day 6 |
| **Adverse reactions after dose 2** | | | |
| Vaccine | 4 | Nasal congestion | Day 28 – Day 29 |
| Placebo | 5 | Body temp 37.5 °C | Day 30 (evening) – Day 31 (morning) |
| Placebo | 6 | Herpes zoster | Day 30 – Day 34 |
| Placebo | 7 | Cough | Day 31 – Day 31 (evening) |
| | | Sore throat | Day 31 – Day 31 (evening) |
| | | Hyperemia arches throat | Day 31 – Day 31 (evening) |
