# Supplementary material for: A Phase 1 Randomized Placebo-Controlled Study to Assess the Safety, Immunogenicity and Genetic Stability of a New Potential Pandemic H7N9 Live Attenuated Influenza Vaccine in Healthy Adults
Source: Vaccines (Basel). 2020 Jun 10;8(2):296. doi: 10.3390/vaccines8020296 (PMC7350028; doi:10.3390/vaccines8020296)
Supplement: Supplementary file 1 [file vaccines-08-00296-s001.zip › Supplementary Table S2.pdf]

Table S2. Laboratory abnormalities in the six days after vaccination or revaccination

Laboratory abnormality	Worst grade	Group			
		Vaccine (n=30)		Placebo (n=8)	
		n[2] (%)	95% CI	n[2] (%)	95% CI
**Dose 1**					
Any laboratory abnormalities[1]	mild	13 (43.3)	27.4–60.8	7 (87.5)	52.9–97.8
Blood chemistry					
Alanine aminotransferase increased	mild	5 (16.7)	7.3–33.6	1 (12.5)	2.2–47.1
Alkaline phosphatase increased	mild	1 (3.3)	0.6–16.7	0	0
Hematology					
Leukocyte count increased	mild	5 (16.7)	7.3–33.6	2 (25.0)	7.1–59.1
Leukocyte count decreased	mild	1 (3.3)	0.6–16.7	2 (25.0)	7.1–59.1
Lymphocyte count increased	mild	5 (16.7)	7.3–33.6	2 (25.0)	7.1–59.1
Lymphocyte count decreased	mild	1 (3.3)	0.6–16.7	0	0
Neutrophil count increased	mild	3 (10.0)	3.5–25.6	2 (25.0)	7.1–59.1
Thrombocyte count increased	mild	0	0	1 (12.5)	2.2–47.1
Erythrocyte count increased	mild	0	0	1 (12.5)	2.2–47.1
Worst grade (total)		mild (n=21)		mild (n=11)	
Any serious adverse event		none		None	
**Dose 2**					
Any laboratory abnormalities[1]	mild	9 (30.0)	16.7–47.9	3 (37.5)	13.7–69.4
Blood chemistry					
Blood bilirubin increased	mild	1 (3.3)	0.6–16.7	1 (12.5)	2.2–47.1
Alanine aminotransferase increased	mild	4 (13.3)	5.3–29.7	0	0
Glucose increased	mild	0	0	1 (12.5)	2.2–47.1
Aspartate aminotransferase increased	mild	1 (3.3)	0.6–16.7	0	0
Hematology					
Leukocyte count increased	mild	4 (13.3)	5.3–29.7	2 (25.0)	7.1–59.1
Leukocyte count decreased	mild	0	0	2 (25.0)	7.1–59.1
Eosinophil count increased	mild	0	0	1 (12.5)	2.2–47.1
Lymphocyte count increased	mild	4 (13.3)	5.3–29.7	2 (25.0)	7.1–59.1
Lymphocyte count decreased	mild	2 (6.7)	1.8–21.3	1 (12.5)	2.2–47.1
Neutrophil count decreased	mild	3 (10.0)	3.5–25.6	1 (12.5)	2.2–47.1
Worst grade (total)		mild (n=19)		mild (n=11)	
Any serious adverse event		none		none	

[1]A subject with more than one abnormal result in a specific category was counted only once. [2]Severity was quantified as follows: none – lack of sign or symptom, normal; mild – events require minimal or no treatment and do not interfere with the subject's daily activities; moderate – events result in a low level of inconvenience or concern to the subject with therapeutic measures; severe – events interrupt the subject's functioning and might require systemic drug therapy or other treatment. [3]Percentages based on total number of subjects in each treatment group.
