# Supplementary material for: A Phase 1 Randomized Placebo-Controlled Study to Assess the Safety, Immunogenicity and Genetic Stability of a New Potential Pandemic H7N9 Live Attenuated Influenza Vaccine in Healthy Adults
Source: Vaccines (Basel). 2020 Jun 10;8(2):296. doi: 10.3390/vaccines8020296 (PMC7350028; doi:10.3390/vaccines8020296)
Supplement: Supplementary file 1 [file vaccines-08-00296-s001.zip › Supplementary Table S3.pdf]

Table S3. Results of RT–PCR testing of volunteers' nasal swabs after immunization with A/17/Hong Kong/2017/75108 (H7N9) LAIV and detection of vaccine virus by culturing nasal swabs in embryonated chicken eggs

Preparation	Volunteer ID Number	Vaccination										Revaccination							
		D1		D2		D3		D4		D5	D6	D1(29)		D2(30)		D3(31)		D4(32)	
		PCR	Eggs	PCR	Eggs	PCR	Eggs	PCR	Eggs	PCR	PCR	PCR	Eggs	PCR	Eggs	PCR	Eggs	PCR	Eggs
**Vaccine**	002	0	0	0	0	0	0	0	0	0	0	+	0	+	+	+	+	+	+
**Vaccine**	003	+	+	0	0	0	0	0	0	0	0	0	0	0	0	0	0	0	0
**Vaccine**	004	+	0	0	0	0	0	0	0	0	0	0	0	0	0	0	0	0	0
**Vaccine**	006	+	0	0	0	0	0	0	0	0	0	0	0	0	0	0	0	0	0
**Vaccine**	007	+	0	0	0	0	0	0	0	0	0	0	0	0	0	0	0	0	0
**Vaccine**	009	+	0	0	0	0	0	0	0	0	0	0	0	0	0	0	0	0	0
**Vaccine**	010	+	0	0	+	0	0	0	0	0	0	0	0	0	0	0	0	0	0
**Vaccine**	012	+	0	+	0	0	0	0	0	0	0	0	0	0	0	0	0	0	0
**Vaccine**	013	+	0	0	0	0	0	0	0	0	0	0	0	0	0	0	0	0	0
**Vaccine**	014	+	+	+	+	+	+	0	0	0	0	0	0	0	0	0	0	0	0
**Vaccine**	015	+	0	0	0	0	0	0	0	0	0	0	0	0	0	0	0	0	0
**Vaccine**	016	+	0	0	0	0	+	+	+	0	0	0	0	0	0	0	0	0	0
**Vaccine**	018	+	0	+	0	+	0	0	0	0	0	0	0	0	0	0	0	0	0
**Vaccine**	019	+	+	+	+	0	0	0	0	0	0	0	0	0	0	0	0	0	0
**Vaccine**	020	+	+	+	0	0	0	0	0	0	0	0	0	0	0	0	0	0	0
**Vaccine**	021	0	0	0	0	0	0	0	0	0	0	0	0	0	0	0	0	0	0
**Vaccine**	022	+	0	+	+	0	0	0	0	0	0	+	0	0	0	0	0	0	0
**Vaccine**	024	+	0	0	0	0	0	0	0	0	0	+	0	0	0	0	0	0	0
**Vaccine**	025	+	0	0	0	0	0	0	0	0	0	+	0	0	0	0	0	0	0
**Vaccine**	027	+	0	0	0	+	0	0	0	0	0	+	0	+	+	0	0	+	+
**Vaccine**	028	+	0	0	0	0	+	0	0	0	0	+	0	+	0	0	0	0	0

Preparation	Volunteer ID Number	Vaccination										Revaccination							
		D1		D2		D3		D4		D5	D6	D1(29)		D2(30)		D3(31)		D4(32)	
		PCR	Eggs	PCR	Eggs	PCR	Eggs	PCR	Eggs	PCR	PCR	PCR	Eggs	PCR	Eggs	PCR	Eggs	PCR	Eggs
**Vaccine**	029	+	0	+	+	+	+	+	+	0	0	0	0	0	0	0	0	0	0
**Vaccine**	030	0	0	0	0	0	0	0	0	0	0	0	0	0	0	0	0	0	0
**Vaccine**	032	+	0	0	0	0	0	0	0	0	0	0	0	0	0	0	0	0	0
**Vaccine**	033	+	0	0	0	0	0	0	0	0	0	0	0	0	0	0	0	0	0
**Vaccine**	035	+	0	0	0	0	0	0	0	0	0	+	0	+	0	0	0	0	0
**Vaccine**	036	+	+	+	0	+	+	0	0	0	0	0	0	0	0	0	0	0	0
**Vaccine**	037	+	0	0	0	0	0	0	0	0	0	+	0	0	0	0	0	0	0
**Vaccine**	039	+	+	0	0	0	0	+	+	+	+	+	+	0	0	0	0	0	0
**Vaccine**	040	+	0	0	0	0	0	0	0	0	0	0	0	0	0	0	0	0	0
**Placebo**	001	0	0	0	0	0	0	0	0	0	0	0	0	0	0	0	0	0	0
**Placebo**	005	0	0	0	0	0	0	0	0	0	0	0	0	0	0	0	0	0	0
**Placebo**	008	0	0	0	0	0	0	0	0	0	0	0	0	0	0	0	0	0	0
**Placebo**	011	0	0	0	0	0	0	0	0	0	0	0	0	0	0	0	0	0	0
**Placebo**	017	0	0	0	0	0	0	0	0	0	0	0	0	0	0	0	0	0	0
**Placebo**	023	0	0	0	0	0	0	0	0	0	0	ARD[1]	ARD	ARD	ARD	ARD	ARD	ARD	ARD
**Placebo**	026	0	0	0	0	0	0	0	0	0	0	0	0	0	0	0	0	0	0
**Placebo**	031	0	0	0	0	0	0	0	0	0	0	0	0	0	0	0	0	0	0
**Placebo**	034	0	0	0	0	0	0	0	0	0	0	0	0	0	0	0	0	0	0
**Placebo**	038	0	0	0	0	0	0	0	0	0	0	0	0	0	0	0	0	0	0
**TOTAL**		**27**	**6**	**8**	**5**	**5**	**5**	**3**	**3**	**1**	**1**	**9**	**1**	**4**	**2**	**1**	**1**	**2**	**2**

\+ (yellow) Detection of vaccine virus by culturing nasal swabs in eggs

\+ (red) Detection of vaccine virus in nasal swabs by RT-PCR

[1]ARD – acute respiratory disease with no connection to influenza. The volunteer #023 was excluded from the trial.
