# Supplementary material for: A Phase 1 Randomized Placebo-Controlled Study to Assess the Safety, Immunogenicity and Genetic Stability of a New Potential Pandemic H7N9 Live Attenuated Influenza Vaccine in Healthy Adults
Source: Vaccines (Basel). 2020 Jun 10;8(2):296. doi: 10.3390/vaccines8020296 (PMC7350028; doi:10.3390/vaccines8020296)
Supplement: Supplementary file 1 [file vaccines-08-00296-s001.zip › Supplementary Table S4.pdf]

Table S4. Stability of attenuating mutations in internal genes of H7N9 LAIV clinical isolates

Isolate number	Day	Gene of L17 MDV origin (attenuating mutation N′t position)							
		**PB2**	**PB1**		**PA**		**M**		**NS**
		**1459**	**819**	**1795**	**107**	**1045**	**68**	**457**	**798**
**Vaccine virus isolation post–Dose 1**									
1	1	+	+	+	+	+	+	+	+
2	1	+	+	+	+	+	+	+	+
3	1	+	+	+	+	+	+	+	+
4	1	+	+	+	+	+	+	+	+
5	1	+	+	+	+	+	+	+	+
6	1	+	+	+	+	+	+	+	+
7	2	+	+	+	+	+	+	+	+
8	2	+	+	+	+	+	+	+	+
9	2	+	+	+	+	+	+	+	+
10	2	+	+	+	+	+	+	+	+
11	2	+	+	+	+	+	+	+	+
12	3	+	+	+	+	+	+	+	+
13	3	+	+	+	+	+	+	+	+
14	3	+	+	+	+	+	+	+	+
15	3	+	+	+	+	+	+	+	+
16	3	+	+	+	+	+	+	+	+
17	4	+	+	+	+	+	+	+	+
18	4	+	+	+	+	+	+	+	+
19	4	+	+	+	+	+	+	+	+
**Vaccine virus isolation post–Dose 2**									
20	29	+	+	+	+	+	+	+	+
21	30	+	+	+	+	+	+	+	+
22	30	+	+	+	+	+	+	+	+
23	31	+	+	+	+	+	+	+	+
24	32	+	+	+	+	+	+	+	+
25	32	+	+	+	+	+	+	+	+
**Control viruses**									
H7N9 LAIV		+	+	+	+	+	+	+	+
L17 MDV		+	+	+	+	+	+	+	+
LWT[2]		–	–	–	–	–	–	–	–

[1]Day of isolation. [2]A/Leningrad/134/57 (H2N2) WT parent of L17 MDV
