# Supplementary material for: A Phase 1 Randomized Placebo-Controlled Study to Assess the Safety, Immunogenicity and Genetic Stability of a New Potential Pandemic H7N9 Live Attenuated Influenza Vaccine in Healthy Adults
Source: Vaccines (Basel). 2020 Jun 10;8(2):296. doi: 10.3390/vaccines8020296 (PMC7350028; doi:10.3390/vaccines8020296)
Supplement: Supplementary file 1 [file vaccines-08-00296-s001.zip › Supplementary Table S5.pdf]

Table S5. Reproductive activity of H7N9 LAIV clinical isolates in developing chicken embryos at various incubation temperatures

Isolate number	Day	Virus titer at specified temperature, $\log_{10}$ EID_{50}/mL			Difference in virus titers at optimal and suboptimal temperature, $\log_{10}$ EID_{50}/mL		Phenotype
		32 °C	26 °C	40 °C	32 °C – 26 °C	32 °C – 40 °C	
**Vaccine virus isolation post–dose 1**							
1	1	7.7	6.5	≤1.2	1.2	*≥6.5*	*ts, ca*
2	1	8.0	6.5	≤1.2	1.5	*≥6.8*	*ts, ca*
3	1	7.8	6.2	≤1.2	1.6	*≥6.5*	*ts, ca*
4	1	7.2	5.8	≤1.2	1.4	*≥6.0*	*ts, ca*
5	1	7.5	5.8	≤1.2	1.7	*≥6.3*	*ts, ca*
6	1	7.2	5.7	≤1.2	1.5	*≥6.0*	*ts, ca*
7	2	8.2	6.5	≤1.2	1.7	*≥7.0*	*ts, ca*
8	2	7.8	6.3	≤1.2	1.5	*≥6.6*	*ts, ca*
9	2	7.7	5.7	≤1.2	2.0	*≥6.5*	*ts, ca*
10	2	7.2	5.8	≤1.2	1.4	*≥6.0*	*ts, ca*
11	3	7.7	5.7	≤1.2	2.0	*≥6.5*	*ts, ca*
12	3	6.9	5.7	≤1.2	1.2	*≥5.7*	*ts, ca*
13	3	8.7	7.5	1.2	1.2	*7.5*	*ts, ca*
14	3	7.6	6.2	≤1.2	1.4	*≥6.4*	*ts, ca*
15	2	7.0	5.8	≤1.2	1.2	*≥5.8*	*ts, ca*
16	3	7.4	6.2	≤1.2	1.2	*≥6.2*	*ts, ca*
17	4	7.4	6.0	≤1.2	1.4	*≥6.2*	*ts, ca*
18	4	9.2	7.4	1.2	1.8	*8.0*	*ts, ca*
19	4	7.4	5.2	1.2	2.2	*6.2*	*ts, ca*
**Vaccine virus isolation post–dose 2**							
20	29	7.8	6.0	≤1.2	1.8	*≥6.6*	*ts, ca*
21	30	8.4	7.2	1.2	1.2	*7.2*	*ts, ca*
22	30	6.9	5.7	≤1.2	1.2	*≥5.7*	*ts, ca*
23	31	8.2	7.0	1.2	1.2	*7.0*	*ts, ca*
24	32	7.7	6.2	≤1.2	1.5	*≥6.5*	*ts, ca*
25	32	7.7	6.5	≤1.2	1.2	*≥6.5*	*ts, ca*
Control viruses							
H7N9 WT		8.7	1.5	9.0	7.2	-0.3	*non–ts, non–ca*
H7N9 LAIV		9.7	7.7	≤1.2	2.0	*≥8.5*	*ts, ca*
L17 MDV		8.0	6.7	1.2	1.3	6.8	*ts, ca*
