# Supplementary material for: A Phase 1 Randomized Placebo-Controlled Study to Assess the Safety, Immunogenicity and Genetic Stability of a New Potential Pandemic H7N9 Live Attenuated Influenza Vaccine in Healthy Adults
Source: Vaccines (Basel). 2020 Jun 10;8(2):296. doi: 10.3390/vaccines8020296 (PMC7350028; doi:10.3390/vaccines8020296)
Supplement: Supplementary file 1 [file vaccines-08-00296-s001.zip › Supplementary Table S6.pdf]

Table S6. Antibody increases in volunteers after vaccination with H7N9 LAIV or placebo, as measured in different assays

Time	Group	n	Number (%) of antibody responses					
			Serum antibody				Nasal sIgA	Total
			HAI	MN	IgA	IgG		
After dose 1 (day 28)	LAIV	30	2 (6.7%)	10 (33.3%)	0 (0.0%)	0 (0.0%)	13 (43.3%)[1]	23 (76.7%)[2]
	Placebo	8	0	0	0	0	0[1]	0[2]
After dose 2 (day 56)	LAIV	30	16 (53.3%)[3]	18 (60.0%)[4]	4 (13.3%)	3 (10.0%)	18 (60.0%)[5]	29 (96.7%)[6]
	Placebo	8	0[3]	0[4]	0	0	0[5]	0[6]
Total conversions	LAIV	30	16 (53.3%)[7]	18 (60.0%)[8]	4 (13.3%)	3 (10.0%)	19 (63.3%)[9]	29 (96.7%)[10]
	Placebo	8	0[7]	0[8]	0	0	0[9]	0[10]

Fisher exact test, two-tailed: 1– $p = 0.0335$; 2 – $p = 0.0001$; 3 – $p = 0.0119$; 4 – $p = 0.0035$; 5 – $p = 0.0035$; 6 – $p < 0.0001$; 7 – $p = 0.0119$; 8 – $p = 0.0035$; 9 – $p = 0.0031$; 10 – $p < 0.0001$.
