# Supplementary material for: A Phase 1 Randomized Placebo-Controlled Study to Assess the Safety, Immunogenicity and Genetic Stability of a New Potential Pandemic H7N9 Live Attenuated Influenza Vaccine in Healthy Adults
Source: Vaccines (Basel). 2020 Jun 10;8(2):296. doi: 10.3390/vaccines8020296 (PMC7350028; doi:10.3390/vaccines8020296)
Supplement: Supplementary file 1 [file vaccines-08-00296-s001.zip › Supplementary Figure S1.pdf]

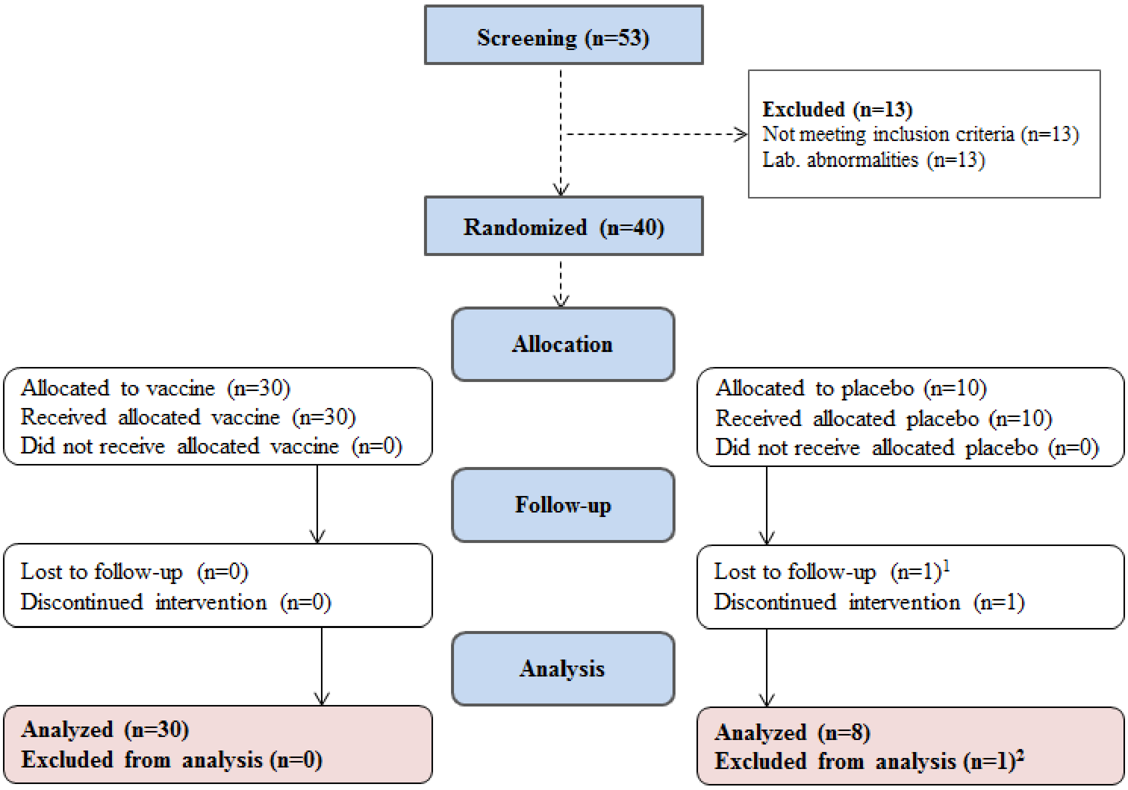

Figure S1. Subject disposition flow diagram. [1]One subject who received the first dose of placebo did not receive the second dose. The subject, a 33-year-old male had increased temperature, cough, headache, fever and rhinitis on day 24. [2]One subject was inadvertently included in the trial, randomized and administered two doses of study placebo. This volunteer was over 50 years old. According to the Protocol ID Number LAIV–H7N9–02, this subject was excluded from the analysis.
