# Supplementary material for: A Phase 1 Randomized Placebo-Controlled Study to Assess the Safety, Immunogenicity and Genetic Stability of a New Potential Pandemic H7N9 Live Attenuated Influenza Vaccine in Healthy Adults
Source: Vaccines (Basel). 2020 Jun 10;8(2):296. doi: 10.3390/vaccines8020296 (PMC7350028; doi:10.3390/vaccines8020296)
Supplement: Supplementary file 1 [file vaccines-08-00296-s001.zip › Supplementary Form S1.pdf]

Form S1. SUBJECT INFORMATION SHEET AND PARTICIPANTS' INFORM CONSENT FORM *B*

**STUDY TITLE:**	REACTOGENICITY, SAFETY AND IMMUNOGENICITY OF A LIVE MONOVALENT A/17/HONG KONG/2017/75108 (H7N9) INFLUENZA VACCINE
**PROTOCOL NO.:**	LAIV-H7N9-02
**SPONSORS:**	Joint-stock company "Scientific and Production Association for Medical Immunobiological Preparations "Microgen"<br>Legal address: 1st Dubrovskaya str., building 15, 115088, Moscow<br>Actual address: 2-nd Volkonsky lane, 10, 127473, Moscow
**INVESTIGATOR:**	Dmitriy A. Lioznov, MD, PhD<br>Smorodintsev Research Institute of Influenza<br>Professora Popova Street 15/17<br>St. Petersburg 197376<br>Russian Federation
**SITE:**	Federal State Budget Institution<br>“Smorodintsev Research Institute of Influenza”<br>Ministry of Health of the Russian Federation<br>Professora Popova str., 15/17<br>St. Petersburg 197376<br>Russian Federation
**STUDY-RELATED PHONE NUMBER(S):**	Marianna K. Erofeeva, MD, PhD<br>Smorodintsev Research Institute of Influenza<br>Ministry of Health of the Russian Federation<br>phone./fax: +7(812) 499.15.37<br>mob.: +7 (905) 265.9109<br>email: erofeeva@influenza.spb.ru (24 hours)

Introduction to the research study

You have completed the screening process for the clinical research study "REACTOGENICITY, SAFETY AND IMMUNOGENICITY OF A LIVE MONOVALENT A/17/HONG KONG/2017/75108 (H7N9) INFLUENZA VACCINE" of the new live flu vaccine (LAIV-H7N9). The results of your medical exams and lab tests confirm that you qualify to be in this study.

An investigational/experimental vaccine is one that is not approved by any regulatory agency nor available to the general population. The goal of the study is to evaluate safety of the vaccine that has been developed against the H7 human influenza virus whereby the vaccine contains a weakened (attenuated) microorganism which is deprived of its virulence leaving only the immunogenic properties. Vaccine is tested in comparison to placebo (substance resembling the real vaccine in appearance and way of application though lacking immunogenic properties). Both the vaccine and placebo are made with chicken embryos. So getting either the vaccine or placebo might cause an allergic reaction or sensitivity to chicken egg protein or other parts of the preparations. Allergic reactions can range from mild to severe. A severe allergic reaction can be life threatening.

If you sign this Inform consent form (*B*) for the research study, then you may participate.

The main goal of a research study is to learn things to help people in the future.

A person who is in a research study is usually called a "subject" (or a "participant" or "volunteer"). A participant of the study has to consent (agree) to be in a research study by signing a consent form.

The study will include 40 healthy adult volunteers who are 18 to 49 years old. The study will last for 56 days (8 weeks), which is just under two months. Further monitoring of your health for 5 months will be held in the form of monthly telephone surveys.

This Inform consent form (*B*) will help you decide if you want to be in this study. Please read this consent form carefully and completely.

If you have any questions about this study or this consent form, you can ask us (the study clinicians). Before signing this consent to participate in the study, you should understand the purpose of this study. You should also understand the procedures to be followed in this study.

Who is paying for this research study?

World Health Organization (WHO) Health Systems and Innovation (HIS) Program Technology Transfer Initiative (TTI) Project is paying for this research study. Currently, WHO holds a leading position in the public health research and supports clinical research aimed at improving the global health system.

Why are we doing this research study?

The aim of the study is to evaluate safety of the vaccine that has been developed against the H7 human influenza virus whereby the vaccine contains a weakened (attenuated) microorganism which is deprived of its virulence leaving only the immunogenic properties. The vaccine is developed against an H7 human influenza virus that might cause severe infection among people. These viruses are considered potentially pandemic and included in the WHO Global Pandemic Preparedness Plan (pandemic is an epidemic of influenza characterized by a more global scale). Effective vaccines against H7 and other flu viruses are a high public health priority around the world because of continued outbreaks of this infection.

Vaccines are the most important way to control and prevent flu and to reduce illness and death caused by the flu. At present, two main types of influenza vaccines are commercially

available, namely inactivated vaccines, which are delivered subcutaneously or intramuscularly and live attenuated vaccines (LAIV), which are given intranasally. Inactivated vaccine product contains no live virus. The viruses included in inactivated vaccines are inactivated so they do not cause influenza, although minor side effects may occur including local reactions at the injection site. LAIV is made from attenuated, or weakened, viruses and does not cause influenza, although it can cause mild signs or symptoms (including rhinorrhoea, nasal congestion, fever or sore throat). Most common side effects from the vaccine are mild and transient compared to symptoms of influenza infection.

The vaccine contains a live influenza H7 virus that has been weakened to remove its ability to cause the flu. Other research studies have strongly suggested that live vaccines are worth exploring because they might have the following advantages over inactivated vaccines:

- Simpler vaccine manufacturing processes with higher yields
- Better immune response to the vaccine in certain populations
- Broader immune responses

Live vaccines are usually given as a nasal spray, so not using needles is another advantage.

In Russia, doctors have used live vaccines for seasonal flu for over 40 years. They proved their effectiveness and safety during this time.

Clinical Study Approval

Approval is required to conduct any clinical study.

The conduct of this clinical study was approved by the Ethics Committee under Ministry of Health of the Russian Federation (Central Ethics Committee). In addition, this clinical trial was approved by the Local Ethics Committee of the institution in which it will be conducted, and by the WHO Ethics Review Committee (ERC).

This study is carried out in accordance with Good Clinical Practice (GCP) as required by applicable rules of the Russian Federation: “Rules of the Quality of Clinical Trials in the Russian Federation”, OST 42-511-1999, and “Good Clinical Practice”, OST 52-379-2005, and the Federal Law “On the Circulation of Drugs” of April 12, 2010, No. 61-FZ.

The study is in line with the World Medical Association Declaration of Helsinki.

How is the study designed?

In this study, we will compare the H7 vaccine to a placebo. A placebo looks like the real vaccine and is given the same way. But the placebo does not have the weakened H7 virus that is needed to make an immune response in the body. In a research study, the researchers use a placebo to help the study results be more objective and to reduce the chance for bias.

Of the 40 healthy adult volunteers who are in the study, 30 will receive study vaccine and 10 will receive placebo. Neither you nor the study clinicians will know which you received. This is called “double blinding.” Like the use of a placebo, a blinded study also helps the study results be more objective and reduces the chance for bias.

Whether you receive the study vaccine or the placebo is decided by chance (like drawing names from a hat). For every four volunteers in the study, three will receive the study vaccine and one will receive the placebo.

We will divide the 40 volunteers into two groups of 20 volunteers each. In each group, 15 people will receive the study vaccine and 5 will receive the placebo.

After the first group receives the first dose of vaccine or placebo, we will observe them for about a week for safety reasons. An independent expert doctor (the study monitor) outside the study will also analyze safety data including any side effects or problems and all lab tests. This independent expert doctor will then advise us if the first group can receive the second dose of vaccine or placebo and the second group can receive the first dose of vaccine or placebo. This independent study monitor will continue to review study information for safety reasons for the rest of the study.

What will happen in the study? (What are the study procedures?)

If you agree to volunteer for the study, you will be in the study for 208 days (about 7 months).

During the study, we will draw blood samples from a vein in your arm. These will be used for lab tests and to check your immune response to the vaccine.

We will also do nasal swab samples to check if the vaccine virus is still growing in your nasal passage. We will do this with cotton swabs with a nylon shaft. We will also collect nasal secrets and saliva samples (with the help of special wicks) to check your immune response to the vaccine.

We will do physical exams that include measuring your temperature, blood pressure, heart rate, breathing rate, and height and weight. On some days you will also have the exam by neurologist to exclude neurologic diseases and an ear, nose, and throat exam.

If necessary on any day, we will also swab near the lower lids of your eyes if you have signs of conjunctivitis (pink or red eye, itchiness, or watery eye).

We will give you the study vaccine or placebo in your nose at two different times.

We will observe you in the isolation unit at the Research Institute of Influenza twice for:

- 7 days after the first vaccination (study Days 0 to 6)
- 7 days after the second vaccination (study Days 28 to 34)

We will provide medical follow-up and monitoring on an out-patient basis during the periods from:

- study Day 7 to Day 27
- study Day 35 to Day 56 (end of the study)

The activities for the study days are described below.

Day 0

- You must come to the study center by 8:00 am fasting.
- We will give you a physical exam including measurement of height and weight, measurement of body temperature, blood pressure, heart rate, respiratory rate, chest auscultation, draw blood for complete blood count, blood chemistry, serology analysis (detection of protective antibodies to influenza viruses) and PBMC analysis (cells responsible for immunity). We will collect blood samples from you in a volume not more than 53 ml (this is about 3.5 tablespoons of blood).
- We will ask you for a urine sample.

- You will also have the exam by neurologist and an ear, nose, and throat exam.
- You will have the exam of Your heart - electrocardiography (ECG), echocardiogram and the study of respiratory function (spirometry).
- We will do a nasal swab one hour before you receive the vaccine or placebo. We will also use small cotton wicks to collect samples from your nose and swabbed sticks to collect saliva samples from your mouth.

The nasal wicks are small tubes of thick cotton with a drawstring attached at one end. You will sit in a comfortable position with your head tilted slightly back. We will insert the wick in your nostril, leave it in for five minutes, and then gently pull it out by the string.

For 30 minutes before the saliva samples, you shouldn't eat, drink, or brush or floss your teeth. You will very gently chew and roll the swab around in your mouth for two minutes, and then carefully spit the swab back into the tube.

- You will receive the first dose of either live influenza vaccine (H7N9) or placebo. Vaccine and placebo will be given in the nose. You will sit in a comfortable position with your head tilted slightly back. We will spray half of each dose (about 0.25 ml) into each nasal passage. You will stay seated with your head tilted slightly back for one minute. We will then observe you for two hours for possible reactions or symptoms.
- You will then enter the clinical isolation unit of the Research Institute of Influenza for almost 7 days (6 nights). We will do one nasal swab each day for the next 6 days.

While you are in the isolation unit, we will carefully observe you and write down any reactions to the vaccination. If you have a reaction, we have equipment and drugs to provide urgent care if you need it.

We will also give you a brief physical exam twice each day (early morning and late afternoon).

Day 3

- We will do a nasal swab, ask you for a urine sample, draw about 6 ml (a little bit more than 1 tea spoon) of blood for complete blood count and blood chemistry, and give you a physical exam.
- You will have the exam of Your heart - electrocardiography (ECG), echocardiogram and the study of respiratory function (spirometry).

Day 6

- We will do a nasal swab, draw about 46 ml (about 3 table spoons) of blood for complete blood count, blood chemistry and PBMC analysis, ask you for a urine sample and give you a physical exam.
- You will also have the exam by neurologist and an ear, nose, and throat exam.
- You will then be discharged from the isolation unit.

After you leave the isolation unit, you will write down all information about your health and any medications you take for the next 3 weeks in a Diary Card. Bring the Diary Card back to your next visit to the study clinic (on study Day 28).

Day 28

- You will return fasting to the clinical isolation unit at the Research Institute of Influenza.
- Women who are able to get pregnant will have a urine pregnancy test.
- We will draw blood for complete blood count, blood chemistry, serology analysis (detection of protective antibodies to influenza viruses) and PBMC analysis (cells responsible for immunity) in a volume not more than 53 ml (this is about 3.5 tablespoons of blood).
- You will also have the exam by neurologist and an ear, nose, and throat exam.
- We will do a nasal swab about one hour before you receive the second dose of vaccine or placebo. We will also use small cotton wicks to collect samples from your nose and swabbed sticks to collect saliva samples from your mouth.
- We will give you a physical exam including measurement of body temperature, blood pressure, heart rate, respiratory rate, chest auscultation.

- After that You will receive the second dose of study vaccine or placebo (with two hours of observation afterwards) and then stay in the isolation unit for 7 days (6 nights). You will have the same procedures and exams that you did during the first isolation unit stay.

Day 31

- We will do a nasal swab, ask you for a urine sample, draw about 6 ml of blood for complete blood count and blood chemistry, and give you a physical exam.
- You will also have the exam of Your heart - electrocardiography (ECG), echocardiogram and the study of respiratory function (spirometry).

Day 34

- We will give you a physical exam including measurement of body temperature, blood pressure, heart rate, respiratory rate, chest auscultation.
- You will have the exam by neurologist and an ear, nose, and throat exam.
- We will draw about 6 ml of blood for complete blood count and blood chemistry, a urine sample for urinalysis and do a nasal swab.

You will then be discharged from the isolation unit. You should again complete your Diary Card for your health and medications for the next three weeks. Bring your Diary Card back to your next visit to the study clinic (on study Day 56).

Day 56 (control study day)

You will return fasting to the study clinic for:

- collection of blood for complete blood count, blood chemistry, serology analysis (detection of protective antibodies to influenza viruses) and PBMC analysis (cells responsible for immunity) in a volume about 3.5 tablespoons of blood;
- collection of nasal swabs and saliva samples.
- Women will have a urine pregnancy test.
- We will give you a physical exam including measurement of body temperature, blood pressure, heart rate, respiratory rate, chest auscultation.
- You will have the exam by neurologist and an ear, nose, and throat exam.

In the next 5 months, your health will be monitored according to the results of a telephone survey on a monthly basis: on Day 88, Day 118, Day 148, Day 178 and Day 208. You will assess your overall well-being on your own, if any serious health changes occur, you will be advised to inform the study site physician by telephone (information is provided in the Informed Consent), if necessary, you will be invited to the study site for examination and consultation.

Is there anything else you should know or do?

During the study, we ask you not to take medications for pain or fever because they can change the test results for the study vaccine or placebo.

Once we admit you to the isolation unit, you are expected to stay there until we discharge you. We will explain the rules and guidelines for the isolation unit, and you are expected to follow them, as well any instructions we give you.

The isolation unit is a comfortable place to stay. It has 14 private rooms with 40 beds (1-4 beds per room), the free space for volunteers' meals (4 times a day) and rest with TV and free wireless internet services.

If you leave the isolation unit before you are discharged, we might have to notify public health authorities for your safety and the safety of those with whom you come in contact.

If during the study You decide to leave the isolation unit while still exhibiting virus shedding the following measures are necessary to be taken and documented:

1) You will be counseled on the potential risk to his (her) isolation unit contacts, as well other hospital and community contacts.
2) You will be recommended strict adherence to standard safety precautions as well as measures to reduce risk of airborne or contact transmission of infectious agents: following rules of individual hygiene, wearing surgical face masks during any community contacts, washing and disinfecting utensils and environmental objects and surfaces.
3) You will be prescribed influenza antiviral oseltamivir treatment for a course of 5 days and provided with appropriate amount of drug.
4) Your nasal specimens should be collected on a daily basis to detect influenza A/H7N9 virus RNA by RT-PCR testing until negative results are obtained.
5) In the case when your contacts show symptoms of influenza like illness, please, immediately inform the study investigators about this fact and inform your community or family contact that nasal specimens should be collected from them to detect influenza A/H7N9 virus RNA by RT-PCR testing until negative results are obtained.
6) If they are PCR positive, they will be prescribed influenza antiviral oseltamivir treatment for a course of 5 days and provided with appropriate amount of drug.

For safety reasons, You will be informed that local public health authorities will be notified of Your premature exiting from the study and the local public health authorities might decide to quarantine You upon condition it is to the benefit of public health. This unlikely event can occur if you are still influenza A/H7N9 virus positive in PCR and there are people from high-risk groups among your close contacts such as pregnant women, young children or immunocompromised persons.

The quarantine is provided in the isolation ward of the clinical trial site or in S.P.Botkin's Municipal Clinical Infectious Diseases Hospital #30 (St. Petersburg).

While you are an out-patient, you can come to the study center during operating hours if you are having side effects or symptoms or you think we should see you.

Vaccine virus might live in your nasal passage for longer than 4 days after each vaccination (this is called virus shedding). We do not expect this to happen.

But to be sure, we will test a swab specimen from your nose to confirm the absence of virus shedding 4 days after each vaccination.

If you show influenza virus shedding on Days 6-7 or Days 34-35 after vaccination, we will ask you to remain in the isolation unit until we detect no influenza virus in your nose for two days in a row. This is for your safety and for the safety of those you might contact.

If you show virus shedding, we might also give you a medicine called oseltamivir that is taken *per os* once a day for 5 days for the treatment of influenza infection.

Women will be required to use reliable birth control methods for the entire study period. We will talk with you about which methods are reliable and also about possible risks if you were to get pregnant during the study. Contraceptives (as appropriate) will be available free of charge to volunteers.

If you get pregnant after you receive the first dose of study vaccine or placebo, you won't be allowed to receive the second dose, and you will be withdrawn from the study.

If you get pregnant after you have received at least one dose of study vaccine or placebo, we will also ask to follow your pregnancy and will report its outcome to the study sponsors (Microgen Company) and to regulatory authorities if required.

We will use your blood samples only for tests needed for this study. We will not use any sample collected as part of the research study phase for HIV, hepatitis, or genetic testing. (Only the sample you provided during screening will be used for HIV and hepatitis testing.)

Once the study is completed, all your blood, urine, saliva, and nasal samples will be destroyed. Samples from the screening process will be destroyed and will not be stored for possible future use.

We can give the results of your study exams and lab tests to your primary health care provider, but only if you want us to do this and agree to it in writing.

Could the study hurt you?

You might have some risks and side effects if you are in the study.

We will spray the weakened vaccine virus for this study into your nose, so it might infect your nasal passage like any influenza virus.

The vaccine was tested in animal studies, and based on study results; we believe it is essentially safe. Besides in previous similar clinical studies of LAIV containing H5N2, H2N2 and H7N3 vaccine viruses no short term and long term safety concerns were raised in

healthy humans. This is the first time that this investigational vaccine will be given to humans. You could have some side effects from the study vaccine.

For example, you might have nasal and other side effects from the vaccination.

Nasal side effects might include

- dry nose
- sneezing
- nose bleed
- a tickling sensation in nasal passages
- nasal congestion
- very runny nose

Other side effects might include

- rise in body temperature
- chills
- confusion
- convulsions/seizures
- fatigue
- sore throat or throat irritation
- rash
- diarrhea
- cough
- difficulty breathing
- painful joints
- vomiting
- muscle aches
- ear pain or discharge
- abdominal pain
- conjunctivitis (swollen eyelids) or pink or red eyes

There might also be risks and side effects of the vaccine that are still unknown. This phase I study is designed to detect only common adverse events and later clinical trials would be needed to confirm the safety profile of this vaccine prior to registration and wider use in the public.

Finally, the virus used for the vaccine is believed to be weakened enough for use in humans. However, it is possible (though unlikely) that it might not be. If this is the case, you might get symptoms of pneumonia. If you have pneumonia symptoms, we will give you the necessary care and treatment.

We will observe you for two hours after you receive the vaccine or placebo and continue to observe you in the isolation unit. If you have an allergic reaction, we have equipment and drugs on hand to treat it.

There is an intensive care unit in the site with all necessary resources, facilities and expertise required for the care of a participant with a serious adverse event. If additional urgent specialized medical care is needed for You being under observation at the Isolation Unit of the Research Institute of Influenza You will be transported to the multidisciplinary S.P.Botkin Municipal Clinical Infectious Diseases Hospital #30 (Mirgorodskaya str. 3, St.Petersburg)

Besides administration of study vaccine or placebo, collection of biological specimens (nasal swab, nasal wick, and blood specimens) may cause some discomfort to subjects. Collection of nasal swab and nasal wick specimens may cause a tickling sensation in the nasal passages during the collection procedures. Venipuncture is sometimes associated with discomfort or pain, redness, swelling, and/or local hardness at the puncture site.

Tell us if you have any side effects, reactions, symptoms, or other problems. Also write them in your Diary Card.

If you have any unexpected or bad symptoms, tell us right away.

If you have been hospitalized or taken to an emergency room during the study, please you or your relatives inform us as soon as possible.

The principal investigator of the research center is responsible for your safety in the course of this study. If something happens to you due to your participation in this study, the principal investigator and his staff will provide you with medical care, the need for which has arisen as a result of the effects of the preparation being studied or manipulations during the study.

Your participation in this study for the entire period of your participation is insured in accordance with the Federal Law No. 61-FZ of April 12, 2010 "On the circulation of Drugs", Government Decree No. 714 "Model Regulations for Compulsory Life and Health Insurance of a Patient Participating in Clinical Trials of Medical Preparations" dated September 13, 2010 (in the latest edition).

Will the study help you?

There are no known benefits to you for being in this research study other than you might get immune protection against the H7 influenza virus.

But you will be helping your country test a new vaccine that could help prevent a future large outbreak of influenza. In addition, new flu vaccines that are easier and faster to produce and potentially less expensive would be a great contribution to global public health.

Will you be paid for being in the study?

Yes, you will be paid for your time for being in the study. This payment is based on a schedule that we will review with you before you agree to be in the study.

If you complete the research study, you will be paid approximately about 57,000 rubles (920 USD) including the tax on personal income (13%). This amount will be divided for each day you participate in the study.

It is possible that we will ask you to remain in the isolation unit longer than planned. If this happens, you will be paid approximately 2280 rubles (40 USD) for each extra day that you are asked to stay.

If you withdraw from the study early, you will be paid for visits and procedures up to the time that you leave the study.

You will be paid on one date in one sum after the study ends. You will also be paid after the study ends even if you left the study early. This payment date will be within one month of the date the rest of your group finishes the study.

Your participation in the clinical study may violate the terms of your existing contract of voluntary medical insurance and deny the right to receive medical care on it.

Will it cost you anything to be in the study?

No, it won’t cost you anything to be in the study. All costs for the study are covered without expense to you.

All volunteers in research studies are guaranteed medical insurance during the study as required by law in the Russian Federation.

But the insurance might not cover expenses for health problems that are not related to your participation in the study.

What if you are hurt or get sick from being in the study?

It is possible that you could be injured or become sick from being in this research study. This might require treatment and result in medical expenses.

If you are injured or get sick, all expenses will be covered under a health insurance policy which is provided to all subjects participating in clinical research studies. The insurance policy is the Policy of Compulsory Life and Health Insurance Coverage for Patients. This is in accordance with the Decree of the Government of the Russian Federation of 13.09.2010 №714: "On Authorization of Standard Rules for Compulsory Life and Health Insurance of Patients Participating in Clinical Studies of Medical Drugs."

The women participating in the study will be counselled on the risks of failure of each method of contraception. If a pregnancy occurs during the trial and leads to either problems during the pregnancy or birth defects, this will be covered by insurance and insurance coverage will be extended.

Will we keep your information confidential?

We will keep your personal identifying information as confidential as possible. We will not share it unless laws and regulations say that it must be shared. We will always try to avoid sharing your name. We will assign you a unique number that we will use to identify you and link you to your study information and samples.

Your medical information from the study will be available to the people and groups listed below who are associated with the study. They include the study clinicians, sponsor representatives, members of National and Local Ethic Committees, and representatives of appropriate regulatory authorities:

- Smorodintsev Research Institute of Influenza
- JSC "Microgen"
- Ministry of Health of Russian Federation
- Independent Ethics Committee (IEC) under Ministry of Health of Russian Federation (Central Ethics Committee)
- Local Ethics Committee at the Research Institute of Influenza
- WHO Ethics Review Committee (This organization oversees the conduct of research clinical studies all over the world).

We can't guarantee absolute confidentiality because of the need to give information to these people and groups connected to the research study.

We won't use your name on your medical information or at any meetings or conferences. We might use the results of the study in research articles. If this is the case, we will never use your name.

If, after signing the informed consent, you decide at some point to refuse to participate in the study, you agree to use the information that was obtained during the study prior to your refusal, on the conditions described above on confidentiality.

By agreeing to participate in the study and signing the informed consent form, you also confirm that you are informed about the processing conditions and allow direct access to your medical information on the conditions described. If you do not agree with these conditions, you have to refuse to participate in the study.

The investigator can give such information respecting regulatory restrictions and ethical considerations only to ethical review committees or similar expert boards or committees, and their affiliated institutions and employees, only under an appropriate understanding of confidentiality with such board or committee

What if we learn about new information while you are in the study?

We will tell you if we learn of any new information that could change your decision to be in the study.

Do you have to be in this study?

No, you don't have to be in this research study. Your participation is completely voluntary. Even if you go through the screening process, you don't have to be in the study.

You can choose not to be in the research study at any time. You do not have to explain why. Leaving the study will not cause you any disadvantages.

If you decide to leave the study early, we will explain how to leave the study as safely as possible.

We also have the right to remove you from the study at any time. This can be done without your consent if we think this is best for you or if you do not comply with the study rules and instructions.

We can remove you from the study for non-compliance if you:

Violate the rules of the isolation unit

Give substantially incorrect or incomplete information on your Diary Card

Skip a scheduled visit

Do not follow instructions at a scheduled visit

Do not tell us about side effects or symptoms

The study sponsor might also decide that the study should stop early. If this happens, we will tell you why the study was stopped.

What are your options if you decide not to be in the study?

This study is being done for research purposes only. It is not meant to provide treatment for an existing medical condition (not related to the study).

Your option is to not be in this research study.

There is no other H7 influenza vaccine currently publicly available in the Russian Federation that you can receive for protection against a future large outbreak of this type of flu.

What if you have questions or want more information?

If you have any questions about the study, including potential risk, side effects or injury, insurance, payment, or your rights as a volunteer, you can contact the study clinician listed below.

Tatiana G. Zubkova, MD, PhD
Head of Clinical Diagnostic Department of
Federal State Budget Institution
“Smorodintsev Research Institute of Influenza”
under Ministry of Health of the Russian Federation
+7 812 499 15 38 (office)
+7 921 316 01 02 (mob.)
You can also notify the Local Ethics Committee at the Research Institute of Influenza regarding anything you think is important about the study. Their phone number is +7 812 499 15 22. This information will be communicated to the medical executive management of the Institute.

Also, you can contact the Ethics Committee under Ministry of Health of the Russian Federation (Central Ethics Committee). Their address is Rakhmanovsky per. 3, Moscow 127994, Russia, and their phone number is +7 495 625 44 21.

You can also notify the Public Joint Stock Insurance Company "Rosgosstrakh", in which you are insured as volunteers. Address: Kievskaya str., 7, 119001, Moscow-59. Contact phone number: +7(495) 783 24 24.

Also you can contact the Sponsor JSC “Microgen”. Address: 2-nd Volkonsky lane, 10, 127473, Moscow, Contact phone number: +7 (495) 790 77 73.

These organizations can help you if you have:

- questions about the study
- questions about your rights as a volunteer for the study
- concerns or complaints about the study

The contact information for the institution conducting the study is listed below:

Federal State Budget Institution
“Smorodintsev Research Institute of Influenza”
under Ministry of Health of the Russian Federation
Prof. Popova str.
15/17, St.-Petersburg
197376, Russia
Phone number and fax: +8 (812) 499 15 00

If you have questions about your rights as a research subject or if you have questions, concerns or complaints about the research, you may also contact:

WHO Ethics Review Committee:
The Secretary of the WHO Ethics Review Committee (ERC)
Dr Abha Saxena – WHO ERC Secretary
ercsec@who.int ; saxenaa@who.int
+41227912406 World Health Organization
Avenue Apia 20, 1211 Geneva, Switzerland

ERC is the organization that conducts independent control of research clinical studies.
ERC will not be able to answer some study-specific questions, such as questions about appointment times etc. However, you may contact ERC if the research staff cannot be reached or if you wish to talk to someone other than the research staff.

INFORM CONSENT FORM

**STUDY TITLE:**	REACTOGENICITY, SAFETY AND IMMUNOGENICITY OF A LIVE MONOVALENT A/17/HONG KONG/2017/75108 (H7N9) INFLUENZA VACCINE
**PROTOCOL NO.:**	LAIV-H7N9-02

My consent for the screening:

MY NAME: ____

DATE OF BIRTH: ____________________

My signature on this consent form means that

1. I have read this consent form (*B*) for participation in the study REACTOGENICITY, SAFETY AND IMMUNOGENICITY OF A LIVE MONOVALENT A/17/HONG KONG/2017/75108 (H7N9) INFLUENZA VACCINE and understood it. I realize that my participation is voluntary.
2. I received information on the goals, objectives and main point of the clinical trial, information about the preparation, its expected benefits, safety and efficacy, the benefits and risks of participating in the clinical trial, and about my rights and responsibilities. I am warned about the possible discomfort, adverse events and my actions in case of unforeseen effects.
3. I have had the chance to ask questions, and I got answers to all of them.
4. I am informed that I will be included in the study if I meet the inclusion criteria for this study.
5. I agree to follow the instructions, voluntarily cooperate with a study investigator and immediately inform him of any violation of my health.
6. I understand that Smorodintsev Research Institute of Influenza under Ministry of Health of the Russian Federation provides insurance in accordance with the Federal Law No. 61-FZ of April 12, 2010 "On the Circulation of Drugs" and the Model Regulations for Compulsory Life and Health Insurance of a Patient Participating in Clinical Trials of Medical Preparations.
7. I am informed that if my health is harmed by direct exposure to the research preparation or by a medical procedure prescribed by the clinical research plan, I will be provided with the necessary medical care, the costs of which will be reimbursed by the insurance company in which I am insured. The amount of compensation can be reviewed by the insurance company in case when my health deterioration occurs due to my non-compliance with the prescriptions of the study investigator.
8. I understand that this study was approved by the Ethics Committee under Ministry of Health of the Russian Federation.
9. I voluntarily and knowingly agree to participate in the clinical study of the medical preparation and understand that I can leave the study at any time without explaining why and it will not cause me any health or legal disadvantages.

10. I understand that any of my primary medical information is available for the study clinician.
11. I understand that my personal identifying information will be kept as confidential as possible.
12. I have been informed that my medical data can be published in medical publications or used in materials for scientific conferences only if I remain anonymous.
13. I understand that some parts of my medical information can be available for the authorized persons or members of National and Local Ethic Committees, and representatives of appropriate regulatory authorities regarding to my participation in the study. I consent to the access of these people to my documentation. I know that they are responsible for protecting private information, and that applicable data protection laws will be respected.
14. I was informed that in accordance with the law "On the Circulation of Drugs" it is prohibited to conduct clinical studies of medicinal products with the participation of law enforcement officers and military personnel. I hereby certify that I do not belong to these categories.
15. I confirm that I received a copy of the volunteer information sheet with the form of informed consent.

Signature ________________________

I received a copy of the volunteer information sheet with the Informed Consent Form *B* on 17 pages. I read it, or somebody read it to me. I understand the information and got answers to all my questions. I agree to participate in the research study on the conditions as indicated above.

____

Surname, name and second name of the subject (legible)

_______________________________________ _________________

Signature Date

I, the undersigned, fully explained this informed consent to the volunteer named above. The volunteer is aware of the participation in the research study, the associated risks and benefits of participation.

____

Study clinician conducting the informed consent process (print name legibly):

_______________________________________ _________________

Signature of the study clinician conducting the informed consent process Date

Individual volunteer identification number (corresponds the number of the policy of clinical trial participant)

There are two copies of this document, one is given to the volunteer, and the second is kept by the investigator.
